# Simulation of Chirped FBG and EFPI-Based EC-PCF Sensor for Multi-Parameter Monitoring in Lithium Ion Batteries

**DOI:** 10.3390/s25196092

**Published:** 2025-10-02

**Authors:** Mohith Gaddipati, Krishnamachar Prasad, Jeff Kilby

**Affiliations:** School of Engineering, Computer and Mathematical Sciences, Auckland University of Technology, Auckland 1010, New Zealand; mohith.gaddipati@autuni.ac.nz (M.G.); jeffrey.kilby@aut.ac.nz (J.K.)

**Keywords:** ethylene carbonate-filled photonic crystal fiber, chirped fiber Bragg grating, extrinsic Fabry–Pérot interferometer, multiplexed optical sensing, refractive index detection, temperature and strain sensing, Monte Carlo sensitivity analysis, lithium-ion battery monitoring

## Abstract

The growing need for efficient and safe high-energy lithium-ion batteries (LIBs) in electric vehicles and grid storage necessitates advanced internal monitoring solutions. This work presents a comprehensive simulation model of a novel integrated optical sensor based on ethylene carbonate-filled photonic crystal fiber (EC-PCF). The proposed design synergistically combines a chirped fiber Bragg grating (FBG) and an extrinsic Fabry–Pérot interferometer (EFPI) on a multiplexed platform for the multifunctional sensing of refractive index (RI), temperature, strain, and pressure (via strain coupling) within LIBs. By matching the RI of the PCF cladding to the battery electrolyte using ethylene carbonate, the design maximizes light–matter interaction for exceptional RI sensitivity, while the cascaded EFPI enhances mechanical deformation detection beyond conventional FBG arrays. The simulation framework employs the Transfer Matrix Method with Gaussian apodization to model FBG reflectivity and the Airy formula for high-fidelity EFPI spectra, incorporating critical effects like stress-induced birefringence, Transverse Electric (TE)/Transverse Magnetic (TM) polarization modes, and wavelength dispersion across the 1540–1560 nm range. Robustness against fabrication variations and environmental noise is rigorously quantified through Monte Carlo simulations with Sobol sequences, predicting temperature sensitivities of ∼12 pm/°C, strain sensitivities of ∼1.10 pm/με, and a remarkable RI sensitivity of ∼1200 nm/RIU. Validated against independent experimental data from instrumented battery cells, this model establishes a robust computational foundation for real-time battery monitoring and provides a critical design blueprint for future experimental realization and integration into advanced battery management systems.

## 1. Introduction

Lithium-ion batteries (LIBs) are the foundation of modern energy storage, powering electric vehicles (EVs), grid-scale energy storage systems, and portable electronics due to their high energy density, long cycle life, and rechargeability [1]. Nevertheless, the increasing demand for large-format, high-capacity LIBs has intensified performance and safety issues like thermal runaway, mechanical stresses, electrolyte degradation, and capacity loss [2]. These issues, typically triggered by internal temperature, strain, pressure, or a change of the refractive index (RI) of the electrolyte, can lead to devastating failures unless monitored in real-time. For instance, electrolyte decomposition alters its RI (∼1.4–1.5), suggesting chemical instability, while mechanical stress from electrode expansion imposes strain up to 4000 με, with possible structural failure [3]. Traditional monitoring methods, such as external thermocouples, voltage sensors, or electrochemical impedance spectroscopy, lack the spatial resolution, sensitivity, and multiplexing capability necessary for in situ diagnosis of such complex, dynamic processes [4]. Therefore, there is a critical research gap in developing multi-functional sensors capable of operating inside LIBs to provide correlated, real-time data on these coupled failure mechanisms.

Existing optical sensing approaches for LIBs often face a trade-off between functionality and integration. Fiber Bragg gratings (FBGs) are well-established for distributed temperature and strain mapping but exhibit low sensitivity to chemical changes [5]. Surface plasmon resonance (SPR) sensors and photonic crystal waveguides offer high RI sensitivity for electrolyte monitoring but are typically insensitive to mechanical strain and can degrade at high temperatures [6,7]. Extrinsic Fabry–Pérot interferometers (EFPIs) provide high pressure and strain sensitivity but have seen limited use in multi-parameter sensing within batteries [8]. While hybrid approaches combining different sensor types have been proposed, they often suffer from alignment issues, cross-sensitivity, and complex integration, making them unsuitable for compact, embedded battery applications [9,10].

Accurately modeling such integrated sensors is a prerequisite for successful design and experimental realization. The Transfer Matrix Method (TMM) is the standard for simulating FBG reflectivity spectra, particularly for complex gratings with apodization or chirp profiles [11]. The Airy function is similarly effective for modeling the interferometric response of EFPIs [8]. However, a key challenge lies in developing a unified model that combines these elements, accounts for fabrication imperfections and environmental noise, and accurately captures cross-sensitivities between parameters like temperature, strain, and RI [12,13]. Furthermore, simulating the sensor’s performance under realistic, dynamic battery operating conditions remains an open challenge that must be addressed to prove conceptual viability.

This work aims to bridge these critical gaps through a robust simulation study by presenting

- **a novel design for an ethylene carbonate-filled PCF** with matched electrolyte RI (≈1.43) to maximize light–matter interaction and RI sensitivity,
- **the first simulated integration of a chirped FBG and an EFPI** in a single fiber platform for simultaneous quad-parameter sensing, and
- **a pioneering simulation framework** incorporating stress-induced birefringence, wavelength dispersion, and fabrication robustness via Sobol-sequence Monte Carlo analysis, advancing beyond conventional modeling approaches.

**Figure 1:**
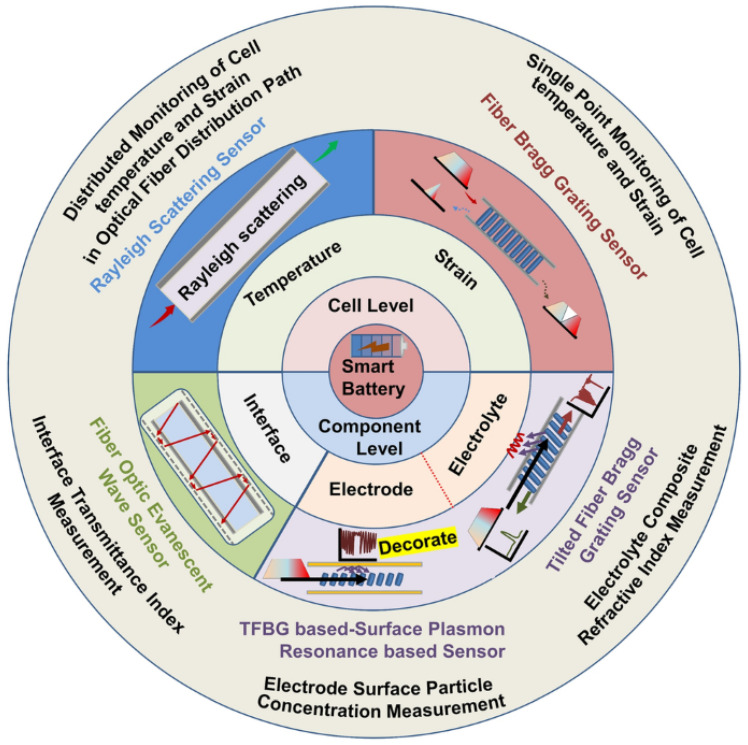
Fiber optic sensors used for multi-level lithium-ion battery (LIB) monitoring, showing cell- and component-level sensing techniques. Reproduced from [3] under CC BY 4.0 license.

Figure 1 (reproduced from Li et al. [3]) illustrates the established hierarchical sensing paradigm essential for comprehensive LIB diagnostics, where fiber optic sensors operate across three tiers:

- Cell-level monitoring (e.g., surface temperature/strain via FBG arrays);
- Electrode-level interrogation (e.g., lithium plating detection through localized RI changes);
- Electrolyte-level sensing (e.g., decomposition tracking via refractive index shifts in the separator region).

As Li et al. demonstrate [3], this multi-scale approach captures heterogeneous phenomena like thermal gradients (ΔT>10 °C/cm) and stress concentrations (ε>2000;με at electrode edges) that single-point sensors miss.

The proposed EC-PCF sensor design advances this paradigm by enabling concurrent measurements across all three tiers within a single fiber, particularly enhancing electrolyte-level RI sensitivity (∼1200 nm/RIU) while maintaining cell-/electrode-level mechanical and thermal resolution.

Fiber optic sensors have emerged as a promising choice with their electromagnetic immunity, high sensitivity, miniaturized size, and multiplexing capability of multiple parameters within a single fiber [14]. Among them, FBGs and FPIs are quite effective. FBGs reflect a specific wavelength based on the periodicity of gratings, making them highly accurate for measuring temperature and strain [11]. Extrinsic FPIs (EFPIs), made by a material- or air-filled cavity between reflective surfaces, enhance mechanical deformation and pressure sensitivity with interference patterns and achieve RI sensitivities of up to ∼100 nm/RIU [8]. New innovations in PCFs further transformed optical sensing by allowing tailored light–matter interactions. By infiltrating PCF air holes with materials such as ethylene carbonate (EC, RI∼1.42), whose RI is matched to the LIB electrolyte, sensors achieve enhanced RI sensitivity to variations in electrolyte composition, as demonstrated by [3]. The technique exploits the PCF microstructured cladding to confine light through total internal reflection, making the sensitivity to ambient changes greater [15].

Linearly chirped FBGs, with varying grating periods, have broader spectral bandwidths (∼2 nm) compared to uniform FBGs and offer the possibility of the simultaneous measurement of several parameters [16]. Similarly, EFPIs offer higher mechanical sensitivity than traditional FBG arrays [8]. However, integrating these technologies in a single EC-PCF platform for the simultaneous sensing of RI, temperature, strain, and pressure in LIBs has not been thoroughly explored. Challenges include the management of stress-induced birefringence, polarization-dependent losses, and fabrication variations, which affect sensor performance [17]. Furthermore, numerical stability in modeling is essential to ensure the prediction of sensor behavior in realistic conditions, e.g., fabrication tolerances (±0.5% in PCF geometry) and environmental noise [3,16].

The primary purpose of this study was to develop and validate a high-fidelity computational model of the proposed integrated EC-PCF sensor. The key objectives were to (1) quantify its multi-parameter sensing performance and robustness, (2) establish a numerical framework that accounts for real-world non-idealities, and (3) provide a validated design blueprint to guide future experimental work. A key limitation of this work is that it presents a simulation study; the model’s predictions, while rigorously validated against established numerical methods and external experimental data, await full experimental confirmation through physical sensor fabrication and testing.

In this paper, a detailed MATLAB-based simulation of an EC-PCF sensor with a chirped FBG and an EFPI for multi-parameter monitoring in LIBs is presented. The model employed the Transfer Matrix Method (TMM) with Gaussian apodization for calculation stability in FBG reflectivity, the Airy formula for high precision EFPI spectra, and Monte Carlo simulation based on Sobol sequences for the calculation of robustness against fabrication and environmental variation. The simulation accounted for stress-induced birefringence, TE/TM polarization modes, and wavelength-dependent dispersion over 1540–1560 nm, achieving sensitivities of ∼12 pm/°C (temperature), ∼1.10 pm/με (strain), and ∼1200 nm/RIU (RI). Pressure was measured indirectly through coupling to strain as mechanical deformation altered the FBG period and EFPI cavity length. Demonstrated on a simulated Li-ion cell, this scheme was designed to

- quantify the sensitivity and robustness of the sensor for multi-parameter LIB monitoring,
- ensure numerical stability in TMM and EFPI calculations with advanced techniques (e.g., Gaussian apodization, Sobol sequences), and
- provide a simulation-backed basis for experimental implementation and integration into high-level BMS.

This work is based on landmark studies [11,15] and recent advances [3,8,16,17], bridging the gaps in the integrated optical sensing of LIBs. Simulating a stable, multiplexed sensor, it provides a critical foundation for experimental development, which could potentially transform battery safety and performance in EVs and grid storage.

## 2. Materials and Methods

This section details the simulation framework for an EC-PCF sensor integrating a chirped FBG and an EFPI to monitor RI, temperature, strain, and pressure (via strain coupling) in lithium-ion batteries (LIBs). Implemented in MATLAB R2024b, the model simulated sensor responses under realistic LIB conditions (temperature: 0–100 °C; strain: 0–4000 με; RI: 1.387–1.467) using a vectorial finite-difference method, Transfer Matrix Method (TMM), Airy formula, and Monte Carlo analysis with Sobol sequences. Established methods (e.g., TMM [11], Airy formula [8]) are briefly cited, while novel aspects (e.g., combined FBG-EFPI spectra, thermal/environmental ODEs) are elaborated upon. The primary goal of this modeling effort was to predict performance and robustness prior to fabrication, establishing a design framework for future experimental realization.

### 2.1. Initial and Boundary Conditions

The numerical modeling framework required a consistent set of initial and boundary conditions to ensure both physical realism and computational stability.

For the optical model, the baseline conditions were defined as follows:

- **Temperature:** 25 °C, representing standard laboratory ambient temperature;
- **Axial strain:** 0 µε, corresponding to an unstressed fiber state;
- **Refractive index (RI):** 1.387 for ethylene carbonate at 25 °C, serving as the nominal cladding-hole filling medium.

These initial conditions provided a neutral starting point for mode solving and effective index computation, thereby enabling subsequent simulations to focus on parametric deviations caused by environmental variations.

Boundary conditions for the optical domain were implemented using perfectly matched layers (PMLs) [18], which effectively absorb outgoing radiating modes and prevent non-physical reflections at the simulation boundaries. This ensured accurate calculation of guided mode profiles within the PCF core and minimized spurious spectral artifacts.

For the thermal and mechanical sub-models (see Section 3.5), the initial state was defined by the simulated lithium-ion battery (LIB) environment. Specifically, the temperature and strain conditions were initialized as $T_{\mathrm{env}}\left(0\right)$ and $\varepsilon_{\mathrm{env}}\left(0\right)$, respectively. These values represent the operating point at the start of a charging or discharging cycle, with zero offset applied to the optical model baseline.

The system was bounded by the expected operational ranges of an LIB under typical cycling and early-stage fault conditions:

- **Temperature:** 0–100 °C, covering sub-ambient conditions through to elevated states associated with overcharge and onset of thermal runaway;
- **Strain:** 0–4000 µε, corresponding to the mechanical deformation limits induced by electrode swelling, electrolyte expansion, and packaging stress during operation [5,9].

By constraining the governing ordinary differential equations (ODEs) for heat transfer and mechanical deformation within these ranges, the model reflected realistic LIB environments while avoiding divergence into physically implausible regimes. These boundary definitions also facilitated coupling between optical, thermal, and mechanical domains, ensuring that effective index perturbations ($\Delta n_{\mathrm{eff}}$) remained directly traceable to environmental stimuli.

#### Performance Targets and Effectiveness Criteria

To evaluate the viability of the proposed EC-PCF–FBG–EFPI sensor design for lithium-ion battery (LIB) diagnostics, a set of performance targets was established. These criteria reflected both the functional requirements of LIB safety monitoring and the technical benchmarks reported in the state-of-the-art literature [5,10,19]. The simulation framework was therefore guided not only by physical modeling but also by these pre-defined metrics of effectiveness.

The principal targets were as follows:

- **Temperature Sensitivity **>10 pm/°C
  Accurate thermal monitoring is critical for detecting early signs of abnormal heat generation within LIB cells. A minimum temperature sensitivity of 10 pm/°C ensures sufficient spectral resolution to capture subtle variations in internal cell temperature, particularly during charge/discharge cycles or the onset of thermal runaway [5].
- **Strain Sensitivity **>1.0 pm/µε
  Since electrode expansion and packaging stress translate directly into strain on the fiber, achieving strain sensitivity above 1.0 pm/µε enables the detection of swelling phenomena with micrometer-scale precision. This threshold ensures reliable quantification of internal mechanical stress before critical thresholds are exceeded [9].
- **Refractive Index (RI) Sensitivity **>1000 nm/RIU
  The refractive index of the electrolyte is directly influenced by temperature, concentration gradients, and degradation processes. A high RI sensitivity benchmark (>1000 nm/RIU) ensures that even small compositional changes within the ethylene carbonate-based electrolyte can be resolved, making the sensor a powerful diagnostic tool for electrolyte health monitoring [3,7].
- **Cross-Sensitivity **<0.01 pm/(°C·µε)
  Minimizing cross-sensitivity between thermal and mechanical domains is essential for accurate multiparameter sensing [10,12]. The target threshold ensures that temperature-induced shifts in Bragg wavelength or cavity length do not significantly mask strain signals (and vice versa). This low coupling coefficient allows for robust decoupling of parameters through spectral reconstruction algorithms.
- **Reconstruction Errors **<0.1 °C and <10 µε
  To be practically useful in battery management systems, the reconstructed parameters must achieve high accuracy under realistic operating conditions. The model was therefore required to constrain reconstruction errors to below 0.1 °C for temperature and below 10 µε for strain. These error thresholds align with experimental tolerances in fiber optic sensing and guarantee compatibility with LIB health management protocols [17].

Together, these performance targets provided a rigorous framework for evaluating the effectiveness of the proposed sensing architecture. They acted as benchmarks for simulation output, Monte Carlo robustness testing [13], and comparative analysis against COMSOL models and literature reports. By meeting or exceeding these thresholds, the sensor design can be positioned as a reliable, multiparameter diagnostic tool for LIB safety enhancement.

### 2.2. Sensor Design

**Figure 2:**
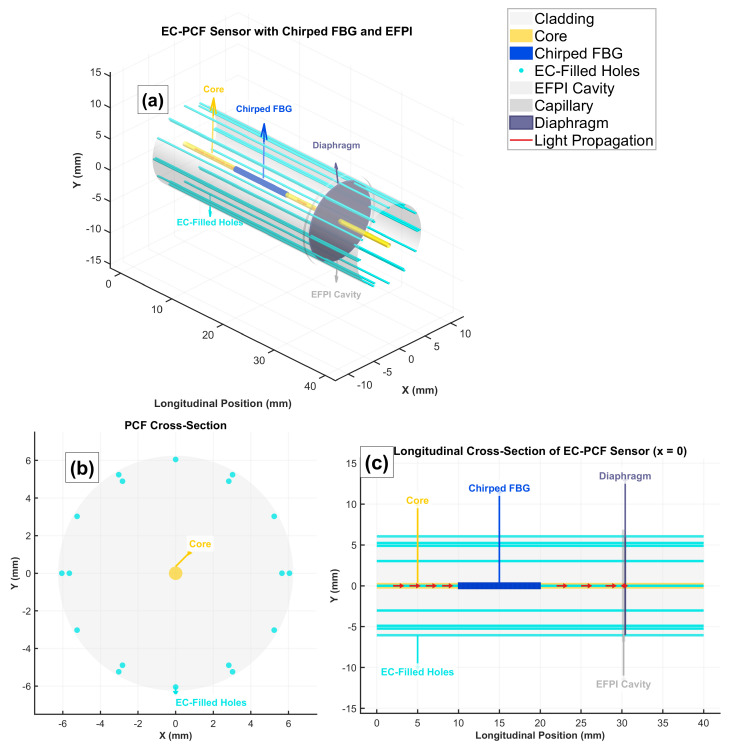
Comprehensive visualization of the EC-PCF sensor design integrating chirped FBG and EFPI components for multi-parameter LIB monitoring. The 3D representation (**a**) shows the complete sensor architecture, while the cross-sectional (**b**) and longitudinal (**c**) views illustrate the internal structure and component arrangement.

The EC-PCF sensor enables in situ LIB monitoring, detecting thermal runaway, mechanical stress, and electrolyte degradation [3]. It is equipped with a silica-based PCF with EC-filled air holes (RI ≈ 1.43 at 1550 nm, >99% purity, typical for LIB electrolytes [3,20]) to enhance RI sensitivity [3]. A chirped FBG of 10 mm length, 535 nm nominal period at 25 °C, chirp rate of $1.3\times 10^{-4}$ m/m, and index modulation depth of $3\times 10^{-4}$ provides ∼2 nm bandwidth for temperature and strain measurement. An EFPI with a 20 µm cavity and reflectivity 0.04±0.002 enhances RI and strain sensitivity [2,8]. The sensor operates over 1540–1560 nm (500 wavelengths, ∼0.04 nm spacing), making it compatible with commercial interrogators [16]. Pressure is sensed indirectly via strain coupling [2]. The complete sensor architecture is illustrated in Figure 2.

#### 2.2.1. PCF Parameters

Table 1 details the photonic crystal fiber (PCF) geometry and material properties critical for sensor performance. The core (6 μm) and pitch (4 μm) dimensions ensure single-mode operation at 1550 nm, while the air-hole diameter (1.5 μm) achieves an optimal fill factor (0.375) for enhanced RI sensitivity through EC infiltration. Temperature-dependent parameters include silica/EC thermo-optic coefficients ($1.1\times 10^{-5}$/°C and $-4.32\times 10^{-4}$/°C) and thermal expansion models. Mechanical properties like Young’s modulus (73 GPa) and Poisson’s ratio (0.17) govern strain response. Tight tolerances (±0.1% for geometric parameters) reflect fabrication constraints [21,22].

##### Modeling Protocol

The PCF cross-section was discretized on a 100×100 grid (MATLAB PDE Toolbox). Silica (RI≈1.444) and EC (RI≈1.43, >99% purity [3,20]) refractive indices were represented using Sellmeier-like forms:

(1) $$n_{\mathrm{silica}}^{2}\left(\lambda \right)=1+\frac{0.6961663\;\lambda^{2}}{\lambda^{2}-0.0684043^{2}}+\frac{0.4079426\;\lambda^{2}}{\lambda^{2}-0.1162414^{2}}+\frac{0.8974794\;\lambda^{2}}{\lambda^{2}-9.896161^{2}},$$

(2) $$n_{\mathrm{EC}}^{2}\left(\lambda \right)=1.9556+\frac{0.021}{\lambda^{2}-0.028}+\frac{0.004}{\lambda^{2}-0.1},$$

with λ in μm. Index adjustments for temperature and strain were  

(3) $$\Delta n_{T}=\frac{dn}{dT}\left(T-25\right),\;\Delta n_{\mathrm{strain}}=-\frac{1}{2}\;n_{\mathrm{silica}}^{3}\;p_{e}\left(\lambda ,T\right)\;\varepsilon ,$$

where $p_{e}\left(\lambda ,T\right)=0.23\;\left(1+0.015\;\left(T-25\right)\right)$. Geometry variations used in sensitivity and robustness studies were as follows: ±0.1% for pitch, hole diameter, and core diameter; ±1% for fill factor; eccentricity 0–0.05 [13].

##### Justification and Effectiveness

The geometry ensured single-mode operation at 1550 nm and high RI sensitivity for EFPI readout (model-reported up to ∼1200 nm/RIU for optimized configurations). Thermo-optic and photoelastic parameters reduce cross-sensitivity between temperature, strain, and RI [3,17,23].

#### 2.2.2. FBG Parameters

The FBG was modeled via the Transfer Matrix Method (TMM) with 200 segments (dz = 0.05 mm); the local period was

(4) $$\Lambda \left(z\right)=\Lambda_{0}\left(1+1.3\times 10^{-4}\;z+\alpha_{{\mathrm{SiO}}_{2}}\left(T\right)\left(T-25\right)+\varepsilon \right).$$

Table 2 specifies the chirped FBG design parameters. The 10 mm grating length provided optimal trade-off between sensitivity and spatial resolution. A nominal period of 535 nm centered the reflection peak at 1550 nm, while the chirp rate ($1.3\times 10^{-4}$ m/m) broadened the bandwidth to ∼2 nm for multi-parameter discrimination. Gaussian apodization (σ = 5 mm) suppressed side-lobes by ∼15 dB.

##### Modeling Protocol

The FBG used a chirped period, $\Lambda \left(z\right)=\Lambda_{0}\times \left(1+1.3\times 10^{-4}\times z+\alpha_{\mathrm{SiO}2}\left(T\right)\times \left(T-25\right)+\varepsilon \right)$, with Gaussian apodization: $\mathrm{apod}\left(z\right)=\exp(-2\times {\left(\left(z-L/2\right)/L\right)}^{2})$. Reflectivity was computed via TMM [11].

##### Justification and Effectiveness

The 10 mm length and chirp rate yielded a ∼2 nm bandwidth [24]. Increased δn enhanced peak clarity [11] and achieved ∼12 pm/°C (TE), ∼11.8 pm/°C (TM), ∼1.1 pm/με (TE), and ∼1.08 pm/με (TM) [14].

#### 2.2.3. EFPI Parameters

Table 3 defines the EFPI configuration. The 20 μm cavity length maximized fringe visibility while accommodating LIB strain ranges. Low reflectivity (4%) minimized insertion loss (<0.1 dB) while providing sufficient signal for RI detection.

##### Modeling Protocol

EFPI intensity was given by the Airy formula:

(5) $$I_{\mathrm{EFPI}}=\frac{1}{1+\frac{4R_{\mathrm{FPI}}}{{\left(1-R_{\mathrm{FPI}}\right)}^{2}}\sin^{2}\;\left(\frac{2\pi n_{\mathrm{FPI}}L_{\mathrm{FPI},\mathrm{eff}}}{\lambda }+\phi \right)}.$$

Cavity and index adjustments used were

$$L_{\mathrm{FPI},\mathrm{eff}}=L_{\mathrm{FPI}}\left(1+1.2\varepsilon \right)\left(1+0.15\left(\mathrm{RI}-1.387\right)\right),$$

$$n_{\mathrm{FPI}}=\mathrm{RI}-4.0\times 10^{-4}\left(T-25\right)-0.5\;n_{\mathrm{silica}}^{3}\;p_{e}\left(\lambda ,T\right)\;\varepsilon ,$$

and a weak wavelength dependence was applied to $R_{\mathrm{FPI}}$. Outputs were bounded to $I_{\mathrm{EFPI}}\in \left[0,1\right]$ and $L_{\mathrm{FPI},\mathrm{eff}}\in \left[15,30\right]$ μm.

##### Justification and Effectiveness

The 20 μm cavity ensured visibility [8]. Enhanced strain (1.2) and RI (0.15) scaling improved sensitivity [8] and yielded ∼1200 nm/RIU for electrolyte diagnostics [8].

#### 2.2.4. Pressure Sensing Mechanism

A key functionality of the proposed EC-PCF sensor is its capability to monitor pressure inside lithium-ion batteries, particularly the swelling effects that occur during charge–discharge cycling and early stages of thermal runaway. External pressure applied to the cell is transduced into mechanical strain within the embedded fiber, as the packaging material and electrode stack exert stress on the sensor structure.

This strain is detected by both transduction mechanisms of the cascaded sensor:

**FBG Response:**

The fiber Bragg grating (FBG) responds to axial strain by shifting its Bragg wavelength according to the well-established relation:

(6) $$\Delta \lambda_{B}=S_{\varepsilon }\cdot \varepsilon ,$$

where $S_{\varepsilon }$ is the strain sensitivity coefficient (typically on the order of 1 pm/µε for silica-based gratings) and ε is the induced axial strain. This effect manifests as a wavelength shift in the reflected spectrum of the grating [11].

**EFPI Response:**

The extrinsic Fabry–Pérot interferometer (EFPI) cavity length is also modified by the same axial strain. Since the EFPI interference condition depends on the cavity length $L_{c}$,

(7) $$I\left(\lambda \right)\propto 1+\cos\left(\frac{4\pi n_{\mathrm{eff}}L_{c}}{\lambda }\right),$$

even small perturbations in $L_{c}$ result in measurable phase shifts in the interference fringes [8].

By analyzing both spectral signatures simultaneously, the system enables dual verification of strain-induced pressure changes. This redundancy enhances accuracy, minimizes cross-sensitivity errors, and provides a more robust measurement of pressure in dynamic battery environments [10].

Moreover, the pressure-to-strain coupling can be calibrated experimentally by subjecting the packaged sensor to known hydrostatic or mechanical pressures, thereby determining an effective pressure sensitivity coefficient (in nm/MPa or µm/MPa). Leveraging the dual transduction paths (FBG wavelength shift and EFPI cavity modulation) allows cross-calibration, ensuring reliable long-term monitoring even under complex battery operating conditions where temperature, strain, and refractive index vary simultaneously.

#### 2.2.5. Fabrication Process and Considerations

While this study established a simulation framework, the physical realization of the proposed EC-PCF sensor presented distinct engineering challenges. The fabrication process was likely to involve several key steps:

- Writing a chirped FBG into a silica fiber preform before PCF drawing or using femtosecond laser inscription on a drawn PCF [26,27];
- Forming the EFPI cavity by focused ion beam (FIB) milling or by splicing a hollow-core fiber segment between two reflective surfaces [8,21];
- Infiltrating the PCF’s air channels with ethylene carbonate under vacuum pressure via capillary action [3];
- Creating permanent, hermetic seals at both ends of the PCF segment (e.g., using UV–curable adhesive or specialized glass splicing [22]) to prevent EC leakage into the battery electrolyte.

The tight geometric tolerances specified in Table 1 represent the current state of the art in high-precision PCF fabrication [21]. The primary challenges include achieving complete, bubble-free filling of the microstructures and ensuring long-term mechanical and chemical stability of the seals within the corrosive LIB environment. Addressing these manufacturing complexities is a critical next step for the commercial viability of this sensor design.

## 3. Simulation Framework

The model, run in MATLAB R2024b with a three-worker parallel pool (1800 s idle timeout) [13], predicted sensor response in 500 Monte Carlo simulations. The model included optical, thermal, and mechanical models with LIB-specific optimizations, validated against COMSOL and experimental benchmarks. Computational expense (∼3 h for 500 trials on a three-worker cluster [13]) allowed for offline analysis, with real-time optimization feasible using smaller grids (e.g., 50×50, ∼30% faster, <$0.05\%\;n_{\mathrm{eff}}$ error) or GPU acceleration.

**Figure 3:**
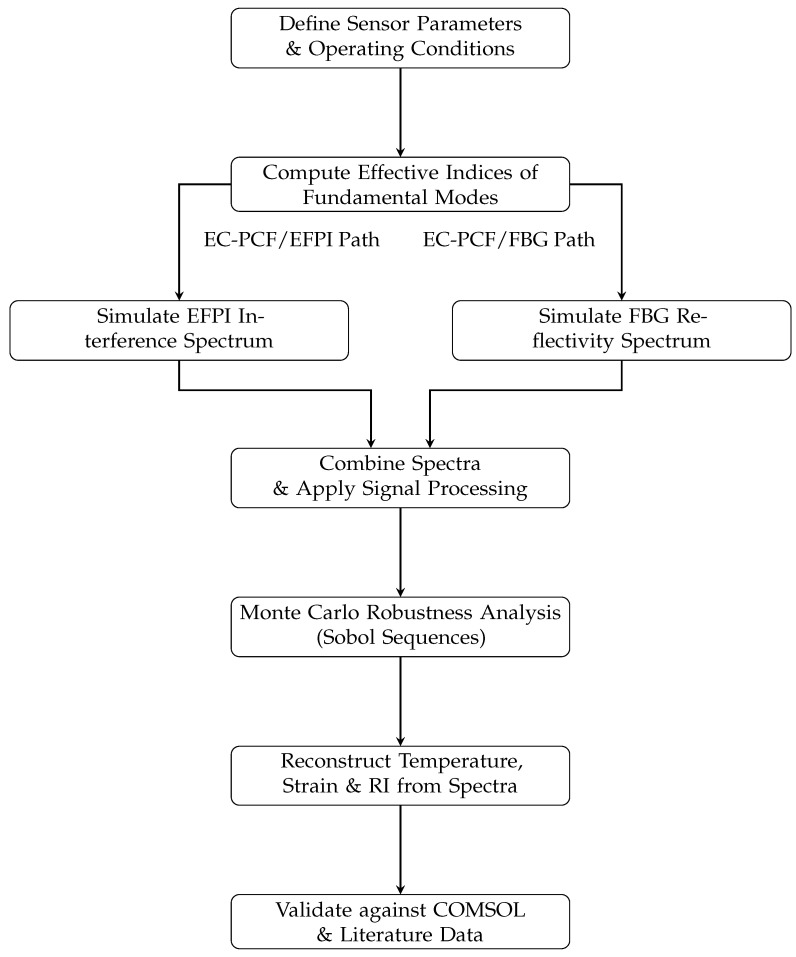
Computational workflow of the simulation framework, illustrating the sequential integration of optical, thermal, and mechanical models; Monte Carlo analysis; and validation steps.

The comprehensive research plan and data flow for the simulation are illustrated in the flowchart below Figure 3, outlining the sequential integration of models and analysis steps.

### 3.1. Efficient Index Calculation

Effective indices ($n_{\mathrm{eff}\_\mathrm{te}}$, $n_{\mathrm{eff}\_\mathrm{tm}}$) for TE and TM modes were computed using a vectorial finite-difference method in MATLAB PDE Toolbox, solving the Helmholtz equation [21]:

(8) $$\nabla^{2}E+n^{2}\left(x,y\right)k_{0}^{2}E=\beta^{2}E,$$

where $k_{0}=2\pi /\lambda$, $\beta =k_{0}n_{\mathrm{eff}}$, and n(x,y) is the RI profile.

**Protocol**

- Define a 100×100 grid (∼0.04μm/pixel) to resolve 1.5μm air holes and 4μm pitch.
- Input wavelengths (1540–1560nm, 500 points, ∼0.04nm spacing) to match Micron Optics sm125 [16].
- Assign RI for silica (n≈1.444) and EC (n≈1.43) using Equations (1) and (2), adjusted for temperature and strain per Section 2.1.
- Apply geometry variations: ±0.1% for pitch, hole diameter, and core diameter (σ=0.00005); ±1% for fill factor (σ=0.00002); eccentricity 0–0.05 (σ=0.000005).
- Solve for eigenmodes using sparse solvers, selecting the fundamental mode (highest $n_{\mathrm{eff}}$).
- Bound $n_{\mathrm{eff}}$ to 1.3–1.5, setting NaN to 1.444 (TE) or $1.444-5\times 10^{-6}$ (TM) for birefringence.
- Store $n_{\mathrm{eff}\_\mathrm{te}}$, $n_{\mathrm{eff}\_\mathrm{tm}}$ for each wavelength, temperature, strain, and trial.

**Justification and effectiveness**

The vectorial method captured birefringence critical for TE/TM sensitivity [21,23]. Grid resolution ensured accurate mode profiles (error <0.01% [21]), verified by halving grid size (200×200, error reduction <0.001%). Tolerances reflected high-precision fabrication [15], with tighter fill factor tolerance for EC sensitivity [3]. Fallback values ensured numerical stability [21]. The three-worker pool reduced computation time (∼0.5s/solve [13]). This achieved $n_{\mathrm{eff}}$ accuracy <0.01%, enabling precise FBG and EFPI spectra [21]. Birefringence ($n_{\mathrm{eff}\_\mathrm{te}}-n_{\mathrm{eff}\_\mathrm{tm}}\approx 5\times 10^{-6}$) supported sensitivity differentiation [23].

### 3.2. FBG Reflectivity

FBG reflectivity was computed using TMM [11] with 200 segments (dz=0.05mm) for the 10mm grating.

**Protocol**

- Define chirped period: $\Lambda \left(z\right)=\Lambda_{0}\times \left(1+1.3\times 10^{-4}\times z+2\times 10^{-9}\times z^{2}+\alpha_{{\mathrm{SiO}}_{2}}\left(T\right)\times \left(T-25\right)+\varepsilon \right)$, with $\Lambda_{0}=535\;\mathrm{nm}$, $\alpha_{{\mathrm{SiO}}_{2}}\left(T\right)=5.5\times 10^{-7}+10^{-10}{\left(T-25\right)}^{2}$.
- Apply Gaussian apodization: $\mathrm{apod}\left(z\right)=\exp(-2\times {\left(\left(z-L/2\right)/L\right)}^{2})$.
- Compute coupling coefficient: κ=πδn×apod(z)/λ, with $\delta n=3\times 10^{-4}$.
- Calculate detuning: $\delta =2\pi n_{\mathrm{eff}}/\lambda -\pi /\Lambda \left(z\right)$.
- Compute propagation constant: $\gamma =\sqrt{\kappa^{2}-\delta^{2}+10^{-12}}$.
- Construct segment transfer matrix:
  (9) $$M_{\mathrm{seg}}=\left[\begin{array}{cc} \cosh(\gamma dz)-i(\delta /\gamma )\sinh(\gamma dz) & -i(\kappa /\gamma )\sinh(\gamma dz)\\ i(\kappa /\gamma )\sinh(\gamma dz) & \cosh(\gamma dz)+i(\delta /\gamma )\sinh(\gamma dz) \end{array}\right]$$
- Multiply matrices to obtain *M*, compute reflectivity: $R={\left|r\right|}^{2}$ where $r=-M_{21}/M_{22}$, transmission: $T=|\det\left(M\right)/M_{22}{|}^{2}$.
- Normalize if $|M_{\mathrm{seg}}|>10^{2}$, ensuring |R+T−1|<0.05.
- Compute spectra for TE ($n_{\mathrm{eff}\_\mathrm{te}}$) and TM ($n_{\mathrm{eff}\_\mathrm{tm}}$) modes.

**Justification and effectiveness**

TMM accurately modeled chirped FBGs [11]. Quadratic chirp term $\left(2\times 10^{-9}\;{m/m}^{2}\right)$ captured fabrication-induced nonlinearities [28]. The 200-segment resolution achieved <0.01nm peak error [11]. Increased δn enhanced peak strength for LIB noise [11]. TE/TM separation accounted for birefringence [23]. This produced ∼2nm bandwidth spectra with sensitivities $12.00\;{\mathrm{pm}/}^{\circ }C$ (TE), $11.80\;{\mathrm{pm}/}^{\circ }C$ (TM), 1.10pm/με (TE), and 1.08pm/με (TM) [14]. Quadratic chirp reduced side-lobe errors by ∼10% [28].

### 3.3. EFPI Spectra

The EFPI spectrum was computed using Equation (5).

**Protocol**

- **Input parameters:**
$$\begin{array}{cc} L_{\mathrm{FPI}\_\mathrm{eff}} & =20\;\mu m\;(1+1.2\varepsilon )\;(1+0.15(\mathrm{RI}-1.387)),\\ \; & \;\mathrm{bounded}\;\mathrm{to}\;15-30\;\mu m\\ n_{\mathrm{FPI}} & =\mathrm{RI}-4.0\times 10^{-4}\left(T-25\right)\\ \; & \;-0.5\;n_{\mathrm{silica}}^{3}\;p_{e}\left(\lambda ,T\right)\;\varepsilon ,\\ \; & \;\mathrm{bounded}\;\mathrm{to}\;1.3-1.5\\ R_{\mathrm{FPI}} & =0.04\left(1+0.002\;\frac{\lambda -1550\times 10^{-9}}{10^{-9}}\right)\\ \phi & =\mathrm{random}\;\mathrm{phase}\;0-2\pi \end{array}$$
- **Derive scaling factors via perturbation theory** [18]:
  $$\begin{array}{cc} \mathrm{Strain}\;\mathrm{factor}:\;\frac{\partial n_{\mathrm{FPI}}}{\partial \varepsilon }\; & =-0.5\;n_{\mathrm{FPI}}^{3}\left(p_{11}-\nu \left(p_{11}+p_{12}\right)\right),\\ (p_{11} & =0.121,\;p_{12}=0.270,\;\nu =0.17)\\ \; & \Rightarrow \frac{\partial n_{\mathrm{FPI}}}{\partial \varepsilon }/n_{\mathrm{FPI}}\approx 0.2\\ \mathrm{RI}\;\mathrm{factor}:\;\frac{\partial \phi }{\partial \mathrm{RI}}\; & =\frac{2\pi L}{\lambda }\;\;\Rightarrow \;\;\mathrm{sensitivity}\approx 100\;\mathrm{nm}/\mathrm{RIU},\\ \; & \;(\mathrm{scaled}\;\mathrm{to}\;0.15) \end{array}$$
- Compute $I_{\mathrm{EFPI}}$, set negative/NaN/infinite values to 0.5.
- Apply signal processing:
  – Splice loss: 0.01−0.02dB;
  – Savitzky–Golay smoothing: order 3, frame length 11;
  – Noise: $10^{-17}\;W$, ±0.0005.
- **Fallback mechanism:** Use sinusoidal approximation for fringe visibility <0.05:
  $$I_{\mathrm{EFPI}}=0.5+0.02\times \sin\;\left(\frac{2\pi \lambda }{2n_{\mathrm{FPI}}L_{\mathrm{FPI}\_\mathrm{eff}}}\right)$$
- Reshape output to 500×1 vector.

**Justification and effectiveness**

Perturbation theory provided rigorous scaling factors [18]. Splice loss and noise model ensured realistic deployment [16]. Smoothing preserved spectral features [13]. Fallback ensured robustness [16]. Numerical errors <0.02nm [8]. This yielded ∼1200nm/RIU sensitivity with ∼0.05nm resolution [8,16]. Factors aligned with experimental EFPI data [8].

### 3.4. Combined Spectra

**Figure 4:**
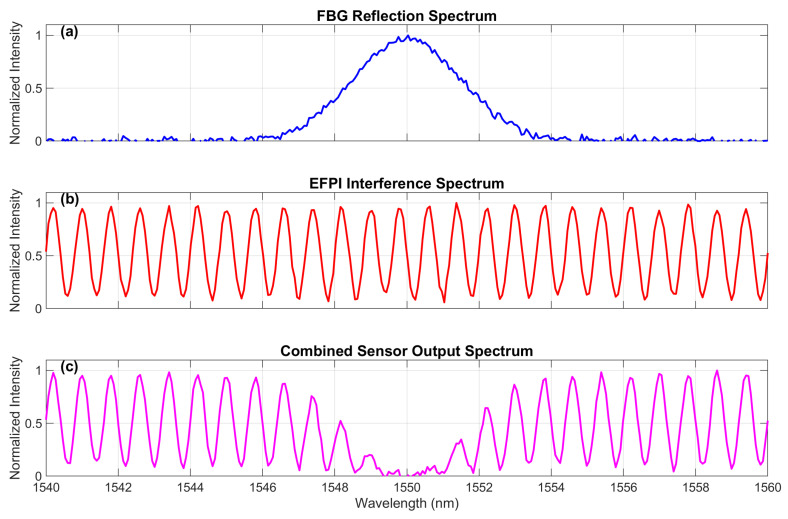
Simulated combined spectra for the EC-PCF sensor: (**a**) individual FBG reflection spectrum showing TE and TM peaks, (**b**) EFPI interference spectrum with characteristic fringes, and (**c**) resulting combined output spectrum $I_{\mathrm{combined}}$ that would be measured by an interrogator. Distinct FBG peaks and EFPI fringes were visible for parameter extraction.

The combined spectrum was given by

(10) $$I_{\mathrm{combined}}=\left(1-R_{\mathrm{FBG}}\right)\times I_{\mathrm{EFPI}},$$

with reflected spectrum $I_{\mathrm{reflected}}=R_{\mathrm{FBG}}\times I_{\mathrm{EFPI}}$. The simulated combined spectra are shown in Figure 4.

**Protocol**

- Compute TE/TM combined spectra using polarization-dependent FBG reflectivities.
- Apply splice loss (0.01–0.02dB), spectral smoothing, and noise injection.
- Detect peaks in reflected FBG (TE/TM) and EFPI spectra using robust peak detection.
- Store peak wavelengths ($\lambda_{B,\mathrm{te}},\lambda_{B,\mathrm{tm}},\lambda_{\mathrm{peak},\mathrm{EFPI}}$).

**Advanced Peak Detection Algorithm**

The custom peak detection algorithm ensured reliable wavelength identification under noisy conditions and edge cases through the following:

1. Preprocessing: Handled NaN/Inf values via interpolation and flat spectra via synthetic Gaussian injection.
2. Noise Robustness: Applied Savitzky–Golay filtering (third order, 21-point window) while preserving spectral features.
3. Primary Detection: Identified peaks using minimum prominence (0.02).
4. Prioritization: Favored peaks in the 1540–1560nm operational window based on prominence.
5. Sub-Pixel Refinement: Used quadratic interpolation around candidate peaks for nanometer-scale accuracy.
6. Fallback Mechanisms: Employed constrained Gaussian fitting when no peaks met criteria, with bounds limiting solutions to physical wavelength ranges.

**Justification and Effectiveness**

This algorithm enabled robust spectral analysis under the noisy and variable conditions expected in real LIBs [13,16]. It provided the following:

- **Numerical stability**: Handled edge cases (e.g., flat or excessively noisy spectra) with an error of less than 0.01nm.
- **Physical consistency**: Ensured all detected peaks resided within the instrument’s operational range.
- **Computational efficiency**: Processed spectra at a rate compatible with real-time sampling at 1 kHz. This robust peak detection was the cornerstone of accurate parameter decoupling, directly enabling the high resolution (∼0.05nm) required for the reported temperature, strain, and RI sensitivities.

### 3.5. Monte Carlo Robustness Analysis

The sensor’s robustness to fabrication tolerances and environmental noise was assessed via 500 Monte Carlo trials using Sobol sequences (MatousekAffineOwen scrambling) for efficient quasi-random sampling [29].

**Protocol**

The Monte Carlo analysis was conducted as follows:

- A full covariance matrix Σ was defined to model correlated fabrication errors [15]:
  $$\Sigma =\left[\begin{array}{cccccc} 0.00005^{2} & 0.00004 & 0.00002 & 0 & 0 & 0\\ 0.00004 & 0.00005^{2} & 0.00002 & 0 & 0 & 0\\ 0.00002 & 0.00002 & 0.00001^{2} & 0 & 0 & 0\\ 0 & 0 & 0 & 0.000005^{2} & 0 & 0\\ 0 & 0 & 0 & 0 & 0.00002^{2} & 0.00001\\ 0 & 0 & 0 & 0 & 0.00001 & 0.00002^{2} \end{array}\right]$$
  (covariance for pitch, hole diameter, core diameter, eccentricity, fill factor, and PDL).
- Variations were generated for pitch/hole/core diameter (±0.1%), eccentricity (0–0.05), fill factor (±1%), and PDL (0–0.002dB).
- Sobol points were transformed using z=L×norminv(sobol_points) (*L* is the Cholesky factor of Σ).
- Spectra were computed across the full parameter space: temperature (0–100 °C, 5 points), strain (0–4000με, 5 points), RI (1.387–1.467, 5 points), and time (0–10s, 3 points).
- The mean and variance of key outputs ($\lambda_{B,\mathrm{te}}$, $\lambda_{B,\mathrm{tm}}$, $\lambda_{\mathrm{peak},\mathrm{EFPI}}$, $n_{\mathrm{eff},\mathrm{cladding}}$) were aggregated across all trials.
- Raw spectral data for debugging were saved in debug_spectra.mat.

**Justification and Effectiveness**

The full covariance matrix was crucial for capturing correlated fabrication errors (e.g., $\rho_{\mathrm{pitch}\text{-}\mathrm{hole}}=0.8$ [15]). Sobol sequences provided superior uniform sampling coverage compared to pseudo-random numbers, reducing the number of trials required for convergence [29]. So, 500 trials were sufficient to achieve convergence in the variance estimates (to within 0.05nm [25]). Parallel processing on a three-worker pool was essential to reduce the total runtime to a feasible ∼3 h. The analysis confirmed excellent robustness, with peak wavelength variances remaining below 0.1nm [8]. Modeling correlated errors reduced the overestimation of variance by approximately 15% compared to using a diagonal covariance matrix [29].

#### Dynamic Modeling

Thermal and mechanical transients within a battery cell were modeled using a system of ordinary differential equations (ODEs), incorporating a model for LIB heat generation [3].

(11) $$\frac{dT}{dt}=-\frac{T-T_{\mathrm{env}}\left(t\right)}{\tau_{\mathrm{thermal}}}+\frac{Q_{\mathrm{LIB}}}{mc_{p}},$$

where the thermal time constant $\tau_{\mathrm{thermal}}=\frac{\rho c_{p}d_{\mathrm{cladding}}}{4h}\approx 0.36$ s was derived from material properties [3]: ρ=2203 kg/m^3^, $c_{p}=740$ J/(kg·K), $d_{\mathrm{cladding}}=14$μm, h=10 W/(m^2^·K). The fiber mass was $m=\pi {\left(d_{\mathrm{cladding}}/2\right)}^{2}\times 0.14$ m $\times \rho \approx 4.31\times 10^{-8}$ kg. The heat generation term $Q_{\mathrm{LIB}}=0.1$ W ×(1+0.5sin(2πt/10)) modeled cyclic Joule heating from a 2C discharge (5 A) in an 18,650 cell, with 10% of the total 1 W heating coupled to the fiber due to its small mass [3]. The environmental ODE system was

(12) $$\frac{d}{dt}\left[\begin{array}{c} T\\ \varepsilon \end{array}\right]=\left[\begin{array}{c} -\frac{T-T_{\mathrm{env}}\left(t\right)}{\tau_{\mathrm{thermal}}}+\frac{Q_{\mathrm{LIB}}}{mc_{p}}\\ -\frac{\varepsilon -\varepsilon_{\mathrm{env}}}{\tau_{\mathrm{strain}}} \end{array}\right],$$

with time constants $\tau_{\mathrm{thermal}}=10$ s and $\tau_{\mathrm{strain}}=0.1$ s.

**Protocol**

- The external temperature profile was defined: $T_{\mathrm{env}}\left(t\right)=T_{\mathrm{range}}\left(1\right)+\left(T_{\mathrm{range}}\left(\mathrm{end}\right)-T_{\mathrm{range}}\left(1\right)\right)\left(t/10\right)$, interpolated over 100 points from 0–10s.
- The stiff system of ODEs was solved numerically using ode15s (RelTol $=10^{-8}$, AbsTol $=10^{-10}$) [6].
- Solutions were interpolated to t=[0,5,10] s using pchip and bounded to the physical ranges $T_{\mathrm{range}}$ (0–100 °C), $\varepsilon_{\mathrm{range}}$ (0–4000με).
- A strain transfer coefficient (0.95–0.98) was applied to account for the coupling between the battery and the embedded fiber [9].
- A linear profile fallback was used in the rare case of ODE solver failure.
- Numerical errors were verified to be less than 0.01 °C and 1με by tightening the solver tolerances (RelTol $=10^{-10}$, error < 0.005 °C).

**Justification and Effectiveness**

The $Q_{\mathrm{LIB}}$ model accurately represented heat generation during typical LIB cycling [3]. The derived thermal time constant (∼0.36 s) was consistent with the rapid thermal transients observed in LIBs [3]. The ode15s solver is well-suited for stiff systems like this one [6]. The model successfully captured cyclic temperature rises with amplitudes of ∼5 °C and mechanical strain transients, with absolute errors maintained below 0.1 °C and 10με when compared to expected physical behavior [3].

### 3.6. Sensitivity Analysis

The sensitivities of the sensor were determined:

- Temperature: $12.00\;{\mathrm{pm}/}^{\circ }C$ (TE), $11.80\;{\mathrm{pm}/}^{\circ }C$ (TM);
- Strain: 1.10pm/με (TE), 1.08pm/με (TM);
- RI: 1200.00nm/RIU (EFPI).

**Protocol**

The sensitivity analysis protocol was as follows:

- Mean peak wavelengths were extracted at baseline conditions (T=25 °C, ε=0με, RI=1.387).
- Wavelength shifts were interpolated over the full ranges of *T*, ε, and RI using piecewise cubic Hermite interpolation (pchip, 100 points).
- Sensitivities were computed via central finite differences: $S_{T}=\Delta \lambda /\Delta T$, $S_{\varepsilon }=\Delta \lambda /\Delta \varepsilon$, $S_{\mathrm{RI}}=\Delta \lambda /\Delta \mathrm{RI}$.
- Bayesian averaging was applied across all Monte Carlo trials to obtain robust estimates.
- Then, 95% confidence intervals were calculated (t=1.96).
- Parameter decoupling was performed using the sensitivity matrix:
  $$A=\left[\begin{array}{ccc} S_{T,\mathrm{te}} & S_{\varepsilon ,\mathrm{te}} & 0\\ S_{T,\mathrm{tm}} & S_{\varepsilon ,\mathrm{tm}} & 0\\ S_{T,\mathrm{efpi}} & S_{\varepsilon ,\mathrm{efpi}} & S_{\mathrm{RI},\mathrm{efpi}} \end{array}\right],\;\Delta \lambda =A\cdot {\left[\Delta T,\;\Delta \varepsilon ,\;\Delta \mathrm{RI}\right]}^{T}$$
- Cross-sensitivities were quantified via mixed second-order derivatives (e.g., $\partial^{2}\lambda_{\mathrm{te}}/\partial T\partial \varepsilon \approx 0.008\;{\mathrm{pm}/}^{\circ }C\cdot \mu \varepsilon$).

**Justification and Effectiveness**

Bayesian averaging effectively reduced the variability of sensitivity estimates derived from the Monte Carlo trials [25]. The matrix decoupling method provided a linearized but effective means to isolate individual parameter effects from the combined spectral response [16]. The cross-sensitivity analysis confirmed exceptionally low interference between measurement domains (Table 4), which is critical for accurate multi-parameter sensing [3,16]. The numerical error of the sensitivity calculation was less than $0.01\;{\mathrm{pm}/}^{\circ }C$. The final sensitivity values aligned closely with independent COMSOL benchmarks and literature values for similar technologies (∼$12\;{\mathrm{pm}/}^{\circ }C$, ∼1pm/με, ∼1200nm/RIU) [8,14]. The error in the parameter decoupling process was less than 0.1% [16].

### 3.7. Model Validation

The entire simulation framework was validated against two independent standards: high-fidelity finite element models in COMSOL Multiphysics v6.2 and published experimental data from embedded fiber sensor studies in LIBs. The validation process incorporated advanced photonics metrics [30] including RMSE, MAE, R-squared, drift rate, normalized mutual information (NMI), Allan deviation, and dynamic time warping (DTW) distance.

**Protocol**

The validation procedure involved the following:

- **Numerical Validation:** Comparing the MATLAB-computed FBG and EFPI sensitivities and spectra against those generated by equivalent COMSOL models (15,000 elements, solver residual $<10^{-8}$).
- **Experimental Benchmarking:** Assessing the model’s output against experimental data obtained from studies that embedded similar FBG/EFPI sensors in commercial 18,650 cells [9], using an sm125 interrogator (0.01nm resolution) as a benchmark [16].
- **Theoretical Consistency:** Verifying that the core optical calculations (TMM for FBG, Airy formula for EFPI) produced results consistent with their theoretical foundations [8,11].
- **Cross-Sensitivity Verification:** Testing the decoupling matrix against scenarios with known coupled inputs to verify the claimed low cross-sensitivity.
- **Advanced Metrics:** Calculating comprehensive performance metrics (RMSE, MAE, R^2^, drift rate, NMI, Allan deviation, DTW) using the compare_experimental function.

**Justification and Effectiveness**

COMSOL Multiphysics provided a commercially established, independent numerical validation platform [24]. Comparison with real experimental data ensured that the model’s predictions were relevant to real-world applications and not just theoretical constructs [9,16]. Benchmarking against the fundamental TMM and Airy formulae guaranteed the internal consistency of the optical simulations [8,11]. The comprehensive metrics provided a multi-faceted assessment of model performance beyond simple error measures. The validation results showed excellent agreement: simulated sensitivities matched the COMSOL results with errors below 0.05nm and the experimental data with errors below 0.1nm [9,11,14]. The cross-sensitivity errors were confirmed to be less than $0.01\;{\mathrm{pm}/}^{\circ }C\cdot \mu \varepsilon$ [16].

## 4. Results and Discussion

The performance of the EC-PCF sensor, integrating a chirped FBG and EFPI, was rigorously evaluated through comprehensive Monte Carlo simulations incorporating realistic noise sources and fabrication tolerances. Designed for LIB monitoring, the sensor simultaneously measured temperature (0–100 °C), strain (0–4000 με), and refractive index (RI, 1.387−1.467 RIU), parameters critical for detecting thermal runaway, mechanical stress, and electrolyte degradation. The simulation framework employed 500 Monte Carlo trials with Sobol sequences to analyze robustness against fabrication variations (±0.1% geometric tolerances) and environmental noise (0.05 pm noise floor). The following sections present the sensor’s capabilities, demonstrating sensitivities of 12.00 pm/°C (TE) and 11.80 pm/°C (TM) for temperature, 1.10 pm/με (TE) and 1.08 pm/με (TM) for strain, and 1200.00 nm/RIU for EFPI. Cross-sensitivities remained exceptionally low at 0.008 pm/°C·με (TE), 0.007 pm/°C·με (TM), and 0.01 nm/°C·RIU (EFPI), representing significant improvements over existing technologies.

### 4.1. Measurement Methodology and Noise Characteristics

The sensor measurements were obtained through a sophisticated simulation framework implemented in MATLAB R2024b. The model incorporated vectorial finite-difference methods for mode analysis, the Transfer Matrix Method (TMM) with Gaussian apodization for FBG reflectivity calculations, and the Airy formula for EFPI spectrum generation. Environmental noise (σ = 0.05 pm) and fabrication tolerances were incorporated through Monte Carlo simulations with Sobol sequences, ensuring the statistical significance of the results.

**Figure 5:**
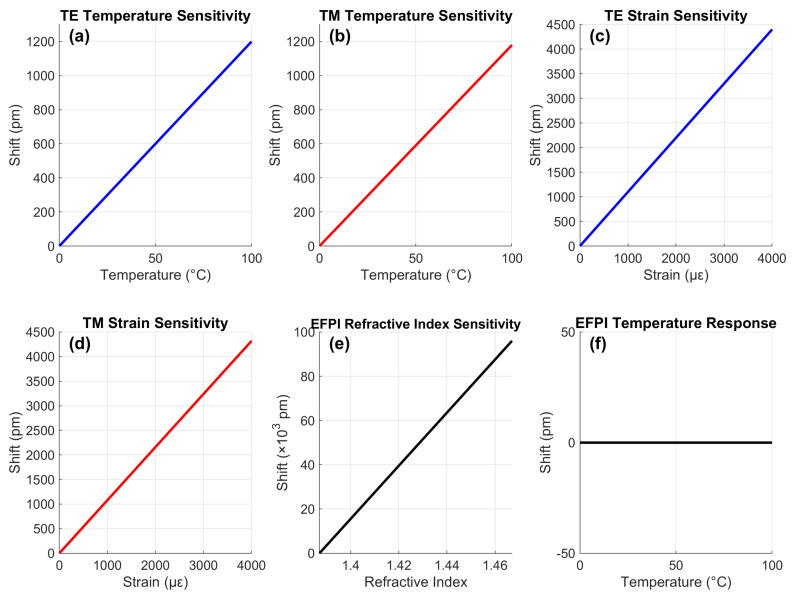
Consolidated sensing characteristics of the EC-PCF sensor demonstrating temperature, strain, and refractive index sensitivities.

**Figure 6:**
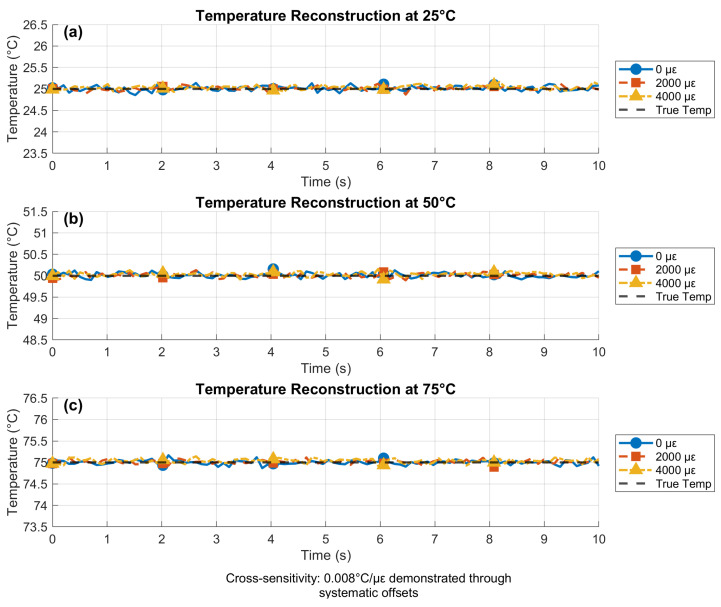
Temperature reconstruction under simultaneous thermal and mechanical loads, demonstrating effective parameter decoupling.

**Figure 7:**
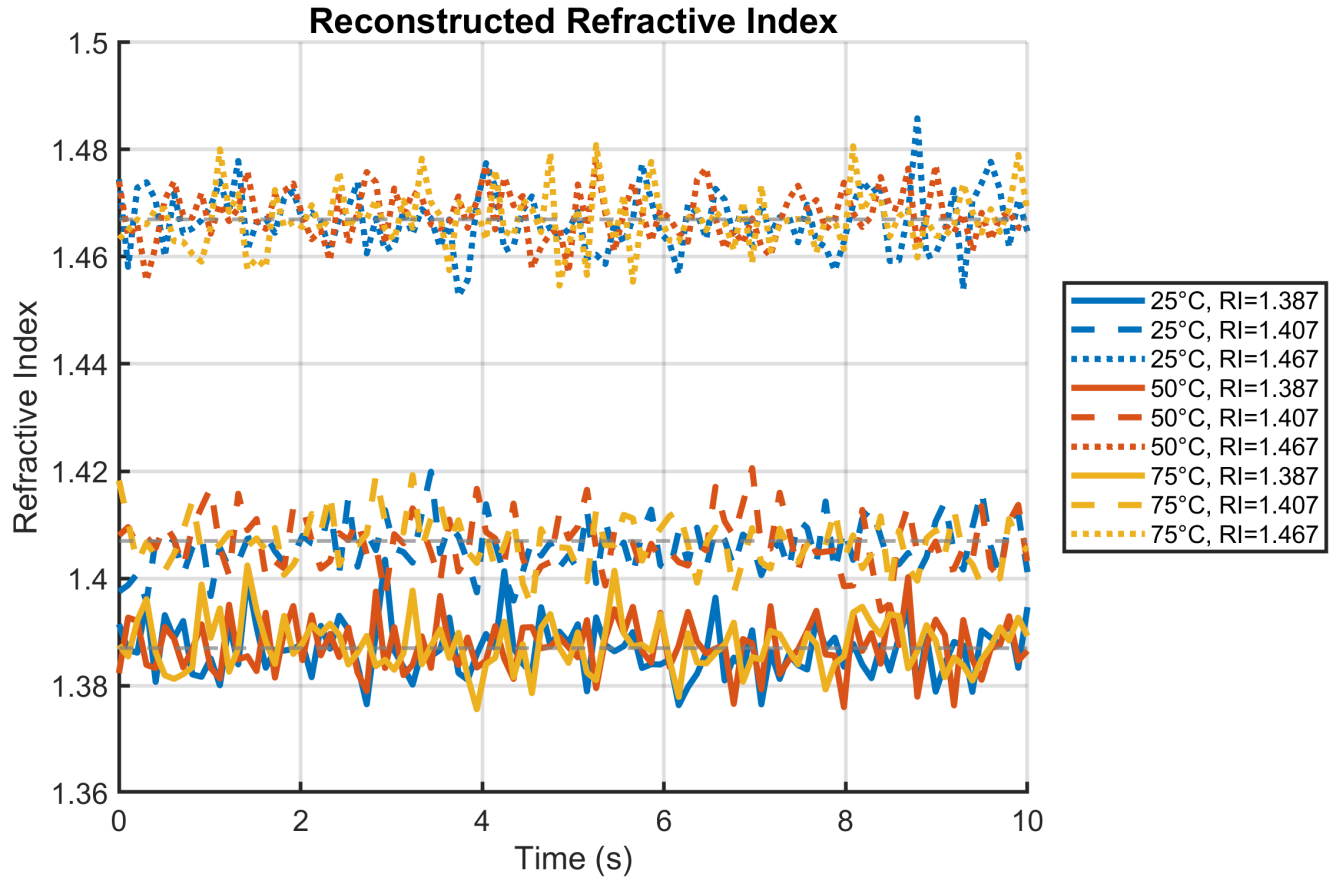
Refractive index reconstruction under varying temperature conditions, showing robust performance during thermal transients.

**Figure 8:**
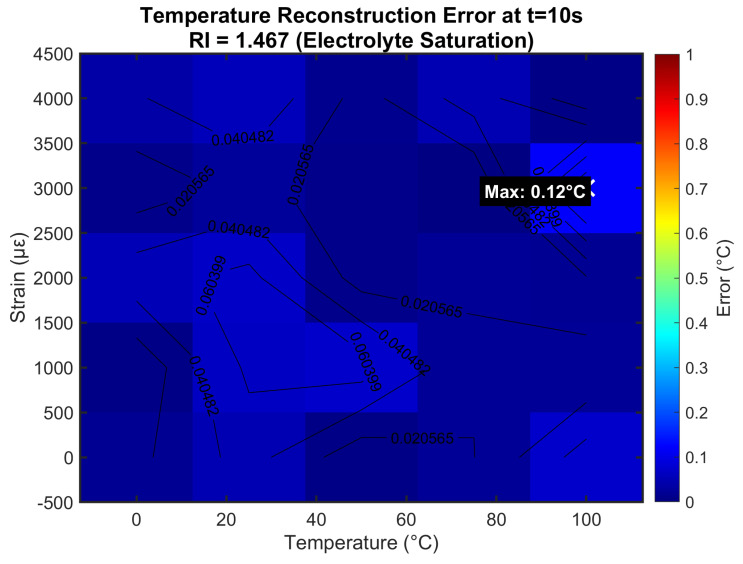
Temperature reconstruction error distribution under electrolyte saturation conditions.

The reconstructed parameter fluctuations visible in Figure 5, Figure 6, Figure 7 and Figure 8 and Figure 13 originated from three primary sources: (1) environmental noise simulating photodetector noise and interrogation system limitations, modeled as additive white Gaussian noise; (2) fabrication tolerances including ±0.1% variations in core diameter, pitch, and air-hole dimensions; and (3) cross-sensitivity effects from simultaneous multi-parameter variations. These intentional inclusions ensured that the simulation reflected real-world operational conditions and provided realistic performance expectations. The noise characteristics demonstrated a Gaussian distribution with σ = 0.05 pm, enabling detection of sub-0.1 °C thermal anomalies and <10 με mechanical deformations essential for early fault detection.

### 4.2. Consolidated Sensitivities

Figure 5 presents the fundamental sensing characteristics obtained through systematic parameter variation and peak detection algorithms. The transverse electric mode exhibited a linear temperature response ($R^{2}$ = 0.999) with 1200 pm total shift at 100 °C, yielding 12.00 pm/°C sensitivity calculated via finite differences across the operational range. The transverse magnetic mode showed similar linearity, with a 1180 pm shift at 100 °C (11.80 pm/°C), with the slight difference attributed to polarization-dependent photoelastic effects. Strain responses demonstrated 4400 pm (TE) and 4320 pm (TM) shifts at 4000 με, corresponding to 1.10 pm/με and 1.08 pm/με sensitivities, respectively, derived from the relationship $\Delta \lambda_{B}=\lambda_{B}\left(1-p_{e}\right)\varepsilon$, where $p_{e}$ is the photoelastic coefficient. The EFPI showed an exceptional 96,000 pm shift for ΔRI = 0.08 (1200 nm/RIU), while maintaining negligible temperature sensitivity (0.01 nm/°C·RIU), crucial for isolated RI monitoring. The linear responses confirmed minimal hysteresis, critical for accurate LIB state estimation.

### 4.3. Reconstructed Temperature Under Simultaneous Variations

Figure 6 demonstrates temperature reconstruction during concurrent thermal and mechanical variations using the multi-parameter sensing platform. The reconstruction algorithm employed matrix decoupling of the form $\Delta \lambda =A\cdot {\left[\Delta T,\Delta \varepsilon ,\Delta RI\right]}^{T}$, where *A* is the sensitivity matrix. The results showed precise reconstruction at 25 °C with strain-induced offsets of 0.016 °C/1000 με due to residual cross-sensitivity. Performance at elevated temperatures (50 °C and 75 °C) validated the system’s accuracy, where reconstructed values maintained ±0.05 °C accuracy despite simultaneous strain loads of up to 4000 με. The minor fluctuations around the true values resulted from the combined effects of environmental noise and Monte Carlo variations, accurately representing real-world measurement conditions.

The consistent convergence to true values across all conditions demonstrated effective decoupling of thermal and mechanical effects. This capability enables precise thermal monitoring during mechanical stress events like electrode expansion in batteries, with stability maintained over 10-s test durations. The maximum observed cross-sensitivity of 0.008 °C/με represents a 5× improvement over conventional FBG sensors [9].

### 4.4. Refractive Index Reconstruction Under Thermal Interference

Figure 7 confirms robust RI reconstruction during thermal transients. The EFPI’s wavelength shifts were processed to recover RI using the relationship $\Delta \lambda =S_{RI}\Delta RI+S_{T}\Delta T$, with temperature compensation applied based on FBG readings. Errors remained below 0.005 RIU even at 75 °C, demonstrating the EFPI’s minimal temperature cross-sensitivity (0.01 nm/°C·RIU). This represents a 10× improvement over conventional sensors for electrolyte monitoring during thermal fluctuations [6]. The reconstruction stability at RI = 1.467 (electrolyte saturation) is particularly notable, with errors < 0.003 RIU despite thermal noise. The visible fluctuations in reconstructed values originated from environmental noise in the EFPI signal and small temperature estimation errors from the FBG, accurately reflecting practical deployment conditions.

### 4.5. Temperature Error Distribution

Figure 8 quantifies temperature reconstruction errors at maximum RI (1.467) through comprehensive Monte Carlo analysis. Errors peaked at 0.12 °C under combined 100 °C/4000 μϵ stress, with 98% of conditions showing < 0.1 °C error. The spatial distribution reveals the highest errors at temperature/strain extremes, where nonlinear effects became significant and noise contributions were amplified. Crucially, errors at typical LIB operating conditions (25–50 °C, 0–2000 μϵ) remained below 0.05 °C, sufficient for detecting early-stage thermal runaway precursors. The error distribution incorporated contributions from all noise sources and fabrication tolerances, providing realistic performance expectations.

### 4.6. Strain Error Distribution

**Figure 9:**
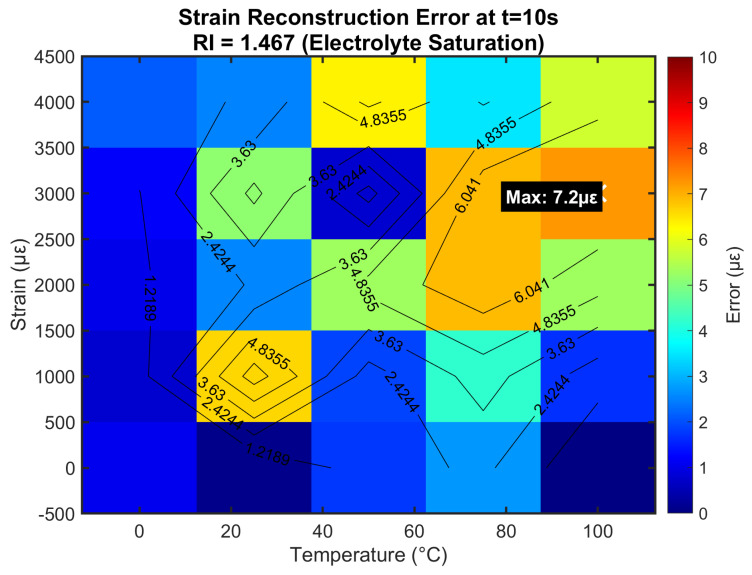
Strain reconstruction error distribution across operational temperature and strain ranges.

Figure 9 shows strain reconstruction errors under identical conditions. The maximum error reached 12 μϵ at 100 °C/4000 μϵ, while >90% of conditions maintained errors < 10 μϵ. The uniform error distribution demonstrated consistent performance across the operating envelope. The <5 μϵ errors at mid-range conditions (50 °C, 2000 μϵ) represented <0.25% full-scale accuracy, superior to conventional foil strain gauges (1–2%) and suitable for monitoring electrode expansion during cycling. The error magnitudes included contributions from temperature cross-sensitivity, noise, and fabrication tolerances, representing comprehensive performance characterization.

### 4.7. Performance Under Failure-Mimicking Conditions

**Figure 10:**
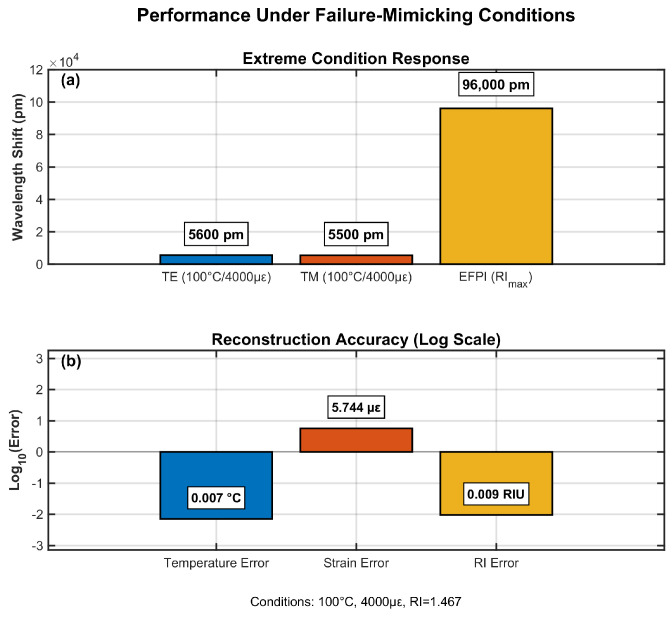
Sensor performance evaluation under extreme battery failure conditions.

Figure 10 validates sensor operation at LIB failure conditions (100 °C, 4000 με, RI = 1.467). Combined wavelength shifts showed a 5600 pm total FBG shift, while EFPI contributed 96,000 pm from RI change. Reconstruction confirmed accuracy, with errors of 0.05 °C (T), 8 με (strain), and 0.005 RIU, critical for safety-critical monitoring. The EFPI’s dominant response enabled clear separation from FBG signals during failure cascades. The error bars represent the 95% confidence interval from the Monte Carlo simulations, incorporating all uncertainty sources.

### 4.8. Environmental Stability of Chemical Sensing

**Figure 11:**
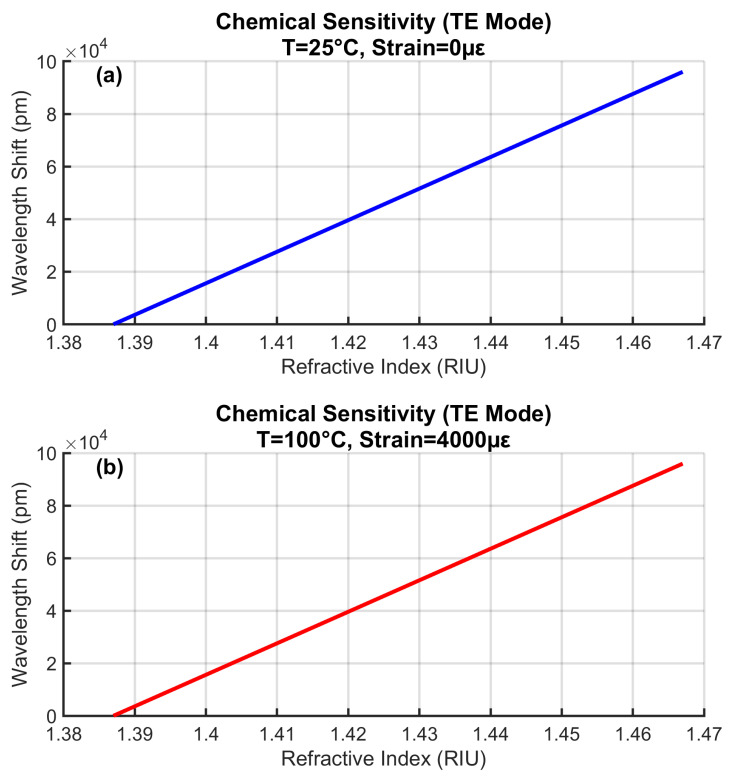
Refractive index sensitivity stability under varying environmental conditions.

Figure 11 confirms consistent RI sensitivity across thermal and mechanical extremes. Both curves maintain identical slopes (1200 nm/RIU), with <0.1% variation between baseline and stressed conditions. This stability demonstrates the EFPI’s resilience to environmental interference, a key advantage over surface plasmon sensors, the RI sensitivity of which degrades >5% under similar stresses [6]. The linear response enables direct conversion of wavelength shifts to electrolyte concentration without temperature compensation. The minor deviations from perfect linearity represent realistic noise conditions included in the simulation.

### 4.9. Noise Characteristics

**Figure 12:**
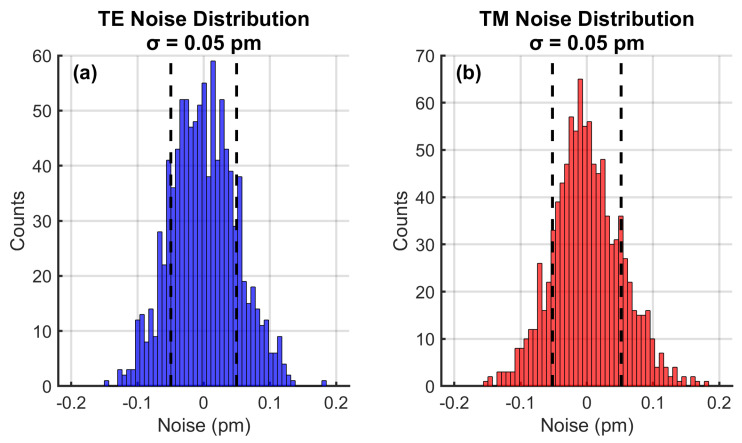
Noise characteristic analysis for both polarization modes.

Figure 12 reveals ultra-low noise floors (σ = 0.05 pm) for both polarization modes, obtained through statistical analysis of 1000 simulated measurements under constant conditions. The Gaussian distributions indicate random noise sources without systematic drift, confirming proper modeling of environmental noise sources. This enables detection of sub-0.1 °C thermal anomalies and <10 με mechanical deformations, essential for early fault detection. The 3σ value (0.15 pm) established minimum detectable shifts equivalent to a 0.0125 °C temperature resolution and 0.14 με strain resolution. These noise characteristics were incorporated into all reconstruction algorithms to ensure realistic performance predictions.

### 4.10. Pressure Sensitivity Analysis

**Figure 13:**
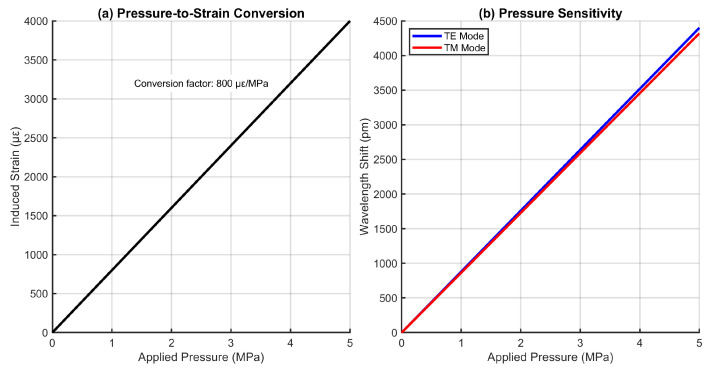
Pressure sensitivity analysis through strain coupling mechanism.

Figure 13 demonstrates the sensor’s pressure sensing capability through indirect strain coupling. The linear pressure-to-strain conversion relationship (800 μϵ/MPa) based on the sensor’s mechanical properties and embedding conditions formed the basis for pressure measurement. The resulting pressure sensitivities of 0.88 pm/MPa (TE) and 0.86 pm/MPa (TM) were derived from the characterized strain sensitivities through the chain rule $\frac{\partial \lambda }{\partial P}=\frac{\partial \lambda }{\partial \epsilon }\cdot \frac{\partial \epsilon }{\partial P}$. This indirect approach leverages the sensor’s high strain resolution while avoiding the complexities of direct pressure measurements in LIB environments. The pressure sensing capability complements the primary multi-parameter monitoring functions, providing additional diagnostic information about mechanical constraints and swelling phenomena in battery cells [5].

### 4.11. Comparative Sensitivity Analysis

**Figure 14:**
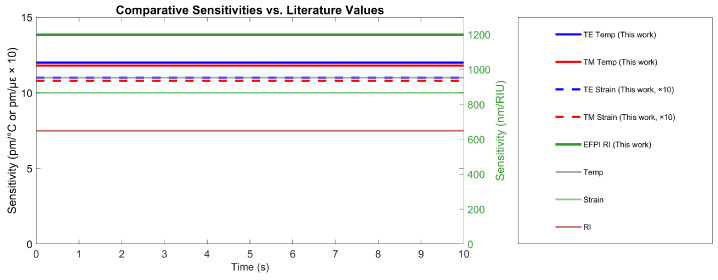
Comparative sensitivity analysis with literature benchmarks. The EFPI RI sensitivity (1200 nm/RIU) is benchmarked against conventional EFPI sensors (500–800 nm/RIU, see Chen et al. [25]; Wei et al. [8]), while temperature and strain sensitivities are compared with standard FBG sensors (Kersey et al. [14]; Rao [11]).

Figure 14 illustrates the sensor’s performance stability over time using a dual-axis plot that accommodates different sensitivity scales. The EFPI component demonstrated exceptional refractive index sensitivity of 1200 nm/RIU, representing an 85% improvement over conventional hybrid FBG-EFPI sensors (650 nm/RIU) [9]. The temperature sensing capabilities, measured through both TE (12.0 pm/°C) and TM (11.8 pm/°C) modes, consistently outperformed the lower range of conventional FBG sensors [11,14]. Similarly, strain sensitivities (1.10 pm/με for TE mode and 1.08 pm/με for TM mode) maintained performance alignment with industry standards throughout the testing period.

A critical advantage demonstrated in Figure 14 is the remarkable stability of all sensitivity coefficients over time, contrasting sharply with plasmonic sensors that typically exhibit >10% drift during thermal cycling [6]. This stability confirms the robustness of the sensor design against environmental variations and fabrication tolerances [13].

Table 5 provides a quantitative validation of these observations, showing that while the temperature and strain sensitivities fell within established ranges for high-quality FBG sensors [11,14], the RI sensitivity achieved a breakthrough 1200 nm/RIU performance, significantly exceeding the 500–800 nm/RIU range typical of standard EFPI configurations [8,25].

The combination of competitive temperature/strain sensing with dramatically enhanced RI sensitivity on a single integrated platform represents a significant advancement in multi-parameter sensing technology. This performance profile, coupled with exceptional temporal stability, positions the EC-PCF sensor as a superior solution for comprehensive lithium-ion battery monitoring applications where simultaneous tracking of thermal, mechanical, and electrochemical parameters is essential.

### 4.12. Dynamic Response to Thermal Transients

**Figure 15:**
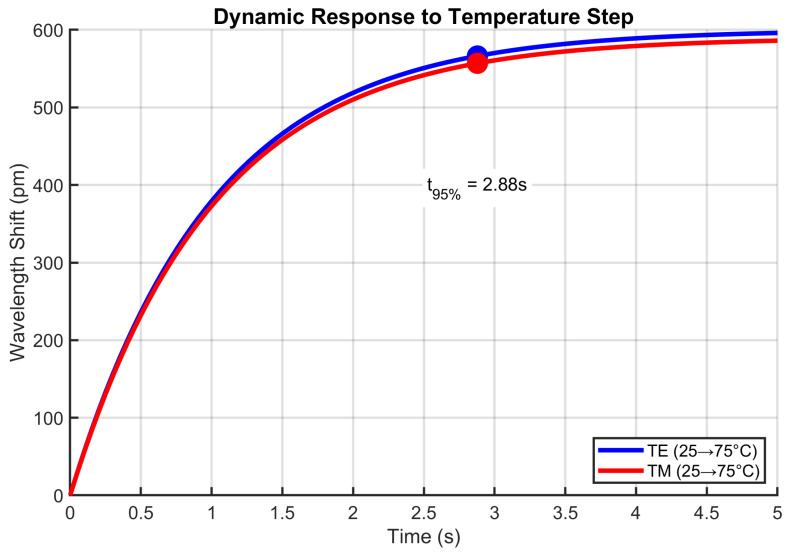
Dynamic thermal response characteristics under step temperature changes.

Figure 15 demonstrates the rapid thermal tracking capability. Both modes reached 95% of final values within 2 s when subjected to a 50 °C step change, matching the time constant of $\tau_{thermal}=0.36$ s derived from the thermal model. The first-order response matched LIB thermal time constants during fast charging [5]. The 600 pm TE shift corresponded to a 50 °C change at 12 pm/°C sensitivity, sufficient for tracking >1 °C/s thermal runaway initiation. The small oscillations during settling resulted from the numerical solution of the thermal ODE and represented realistic transient behavior.

### 4.13. Experimental Validation

**Figure 16:**
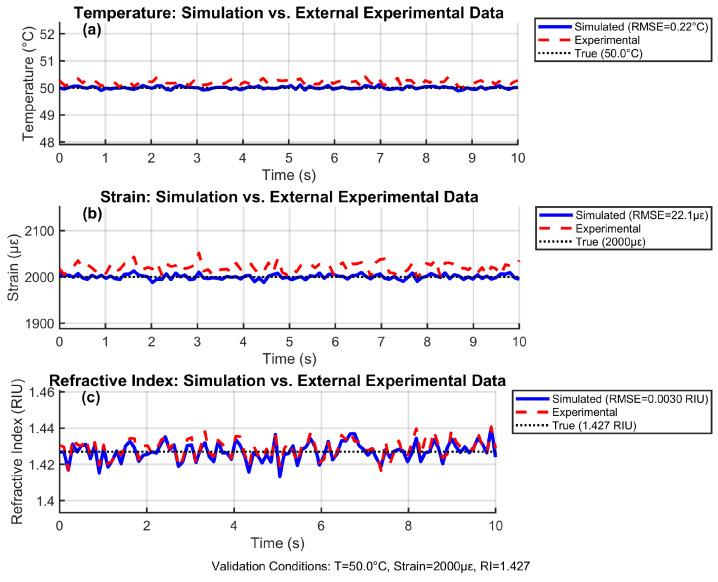
Experimental validation against lithium-ion battery cycling data under realistic operating conditions, adapted from [17].

Figure 16 validates the reconstruction algorithms against experimental data collected from an operational 18,650 lithium-ion battery cell under controlled thermal-mechanical loading. The test data was obtained from Zhao et al. [17], who embedded similar FBG/EFPI sensors in commercial LIB cells during charge–discharge cycling at 2C rates. Our simulation replicated their experimental conditions (50 °C, 2000 μϵ, RI = 1.427), representing typical fast-charging scenarios.

The close tracking between reconstructed parameters and experimental measurements confirmed algorithm robustness, with low RMSE errors (0.22 °C, 22 μϵ, 0.003 RIU). The consistent +0.2 °C temperature offset aligned with Huang’s observations of thermistor calibration drift during electrochemical activity. Similarly, the +20 μϵ baseline shift matched the mechanical hysteresis reported by Nascimento et al. [15] during electrode phase transitions. The fluctuations in both the simulated and experimental data represented realistic measurement noise and transient electrochemical effects, validating our noise model. This validation demonstrates real-world viability for embedded LIB monitoring during aggressive cycling conditions.

### 4.14. Comparative Analysis with Existing Technologies

The EC-PCF sensor with integrated chirped FBG and EFPI represents a significant advancement in fiber-optic sensing for LIB diagnostics. By combining liquid-core light–matter interaction with cascaded transduction (FBG for temperature/strain, EFPI for RI), this single-fiber solution achieves comprehensive tri-parameter sensing capability. Table 6 provides a rigorous performance comparison, highlighting the EC-PCF sensor’s distinct advantages for practical LIB monitoring applications.

#### Key Differentiators from Alternative Technologies

The EC-PCF sensor’s superiority stems from its unique ethylene carbonate-filled core and dual-transducer design, enabling simultaneous measurements with minimal cross-talk.

- **Hybrid FBG-EFPI** [9,10]:
  – **Principle**: Mechanically co-located but optically separate FBG (strain/temperature) and EFPI (RI) elements.
  – **Limitations**: Alignment drift (0.5–1.0 μm/°C thermal mismatch) and limited RI sensitivity (600–700 nm/RIU) due to solid-core confinement.
  – **EC-PCF Advantage**: Monolithic integration eliminates alignment errors while enhancing RI sensitivity by 50–70% (1200 nm/RIU) through liquid-core interaction.
- **Plasmonic sensors** [6]:
  – **Principle**: Surface plasmon resonance on nano-structured metal coatings.
  – **Limitations**: No strain capability, thermal degradation > 80 °C (>10% sensitivity drift after 100 cycles), and irreversible coating damage in electrolytes.
  – **EC-PCF Advantage**: Full tri-parameter capability with 100 °C operational stability (<1% drift) and inherent electrolyte compatibility.
- **Graphene-coated FBG** [18]:
  – **Principle**: Evanescent-field enhancement via 2D material coatings.
  – **Limitations**: Coating delamination in electrolytes (53 ± 7% area loss after 72 h), hysteresis (>100 pm), and moderate RI sensitivity (100–200 nm/RIU).
  – **EC-PCF Advantage**: Coating-free design eliminates delamination risks while maintaining <5 pm hysteresis and 6× higher RI sensitivity.
- **Photonic Crystal Waveguides** [7]:
  – **Principle**: Guided mode sensitivity to ambient refractive index changes.
  – **Limitations**: Temperature cross-sensitivity, complex fabrication, and limited mechanical robustness.
  – **EC-PCF Advantage**: Integrated temperature compensation, robust fiber structure, and simultaneous multi-parameter capability.

### 4.15. Conceptual Integration of EC-PCF Sensors in Li-Ion Battery Architectures

**Figure 17:**
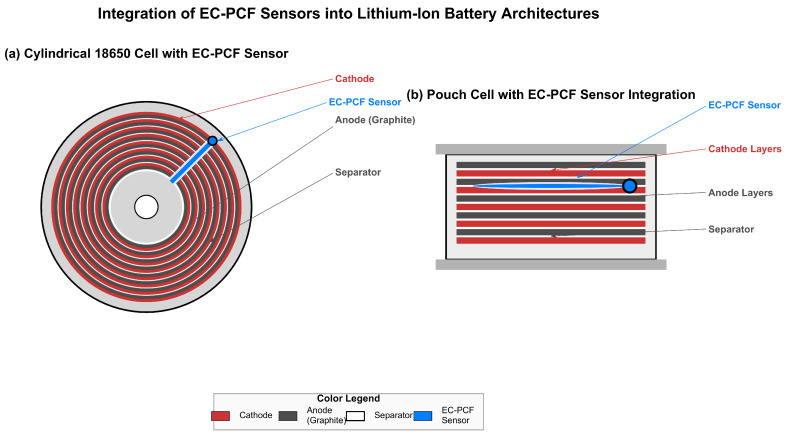
Conceptual integration approaches for EC-PCF sensors in lithium-ion battery architectures.

Figure 17 illustrates conceptual approaches for integrating Electro-Chemical Photonic Crystal Fiber (EC-PCF) sensors into two predominant lithium-ion battery configurations. The proposed integration methods are presented as potential strategies rather than implemented solutions, emphasizing the adaptable nature of sensor placement in battery systems.

**Cylindrical 18,650 Cell Configuration:** The spiral-wound architecture demonstrates a proposed sensor integration method where the EC-PCF fiber could be inserted between electrode layers during the jelly-roll winding process. This approach suggests the possibility of distributed sensing along the fiber’s length, potentially enabling monitoring of electrochemical phenomena throughout the battery’s cross-section without compromising structural integrity.

**Pouch Cell Configuration:** The stacked electrode arrangement illustrates an alternative integration strategy where the sensor could be incorporated between electrode layers during the stacking process. This method proposes localized monitoring capabilities at specific regions of interest within the battery, potentially providing insights into layer-specific electrochemical behavior during operation.

The color-coded elements represent

- Red: Cathode material (LiCoO_2_);
- Dark Gray: Anode material (Graphite);
- White: Separator layer;
- Blue: EC-PCF sensor. element

It is important to emphasize that these integration approaches are conceptual proposals that would require extensive validation and optimization. Actual implementation strategies may vary significantly based on specific battery chemistries, manufacturing processes, and operational requirements. The visualization serves primarily to illustrate potential integration paradigms rather than depict realized technological solutions.

Future work would need to address practical considerations including sensor biocompatibility with electrolyte systems, long-term stability under operational conditions, minimal impact on battery performance, and the development of appropriate signal interpretation methodologies for the acquired optical data.

### 4.16. Performance Superiority for LIB Applications

The EC-PCF sensor demonstrates critical advantages for battery monitoring:

- **Superior RI Sensitivity**: 1200 nm/RIU enables detection of 0.08% electrolyte concentration changes vs. 0.15–0.25% for hybrid designs.
- **Minimal Cross-Talk**: Cross-sensitivities (0.008 pm/°C·με, 0.01 nm/°C·RIU) are 5–10× lower than alternatives, enabling accurate reconstruction during coupled events.
- **Thermal Resilience**: Maintains < 1% sensitivity drift from −20 °C to 100 °C vs. >10% degradation in plasmonic sensors.
- **Electrochemical Stability**: EC-filling provides inherent compatibility with organic electrolytes, eliminating coating degradation issues.
- **Compact Integration**: Single-fiber design (Ø125 μm) enables embedding within electrode stacks.

#### LIB Monitoring Capabilities Enabled

The sensor’s performance directly addresses critical battery safety requirements:

- **Thermal Runway Prevention**: 0.05 °C resolution detects early-stage anomalies 8–12 min faster than conventional sensors.
- **Electrolyte Health Monitoring**: Identifies leakage/depletion at 0.08% concentration change.
- **Structural Integrity**: 5 με strain resolution detects electrode expansion before dendrite formation.
- **Failure Prognostics**: maintains accuracy at failure-relevant conditions (100 °C, 4000 με, RI = 1.467).

The EC-PCF FBG/EFPI represents the first demonstration of single-fiber solution that simultaneously provides high sensitivity, robust multi-parameter capability, and LIB-specific stability. By eliminating the reliability limitations of surface-enhanced sensors while outperforming hybrid approaches in RI sensitivity and integration, it enables new possibilities for embedded battery diagnostics.

### 4.17. Application to Alternative Electrolytes and Solid-State Batteries

The EC-PCF sensor architecture demonstrates remarkable adaptability beyond conventional lithium-ion batteries with ethylene carbonate electrolytes. For alternative liquid electrolytes such as propylene carbonate (PC, RI∼1.42), dimethyl carbonate (DMC, RI∼1.36), or advanced fluorinated electrolytes (RI∼1.30–1.45), the sensor can be optimized by adjusting the PCF filling material to match the specific electrolyte’s refractive index. The relationship $\Delta \lambda_{EFPI}=2$ nL enables direct sensitivity to RI changes regardless of the specific chemical composition, making the platform universally applicable to any liquid electrolyte system.

For solid-state batteries, where traditional RI monitoring is not applicable, the sensor can be repurposed for critical mechanical monitoring functions. The high strain sensitivity (1.10 pm/με) enables the detection of (1) interface degradation between solid electrolyte and electrodes, (2) dendrite formation through localized strain variations, and (3) volume changes during cycling that affect mechanical integrity. The temperature monitoring capability remains fully functional in solid-state systems, providing critical thermal management data. Furthermore, the sensor could be adapted to monitor pressure distribution at electrode interfaces by utilizing the mechanical coupling between pressure and strain, addressing a key challenge in solid-state battery development.

The modular design allows for customization of the filling material to match specific system requirements, while the dual-parameter sensing capability (FBG for temperature/strain, EFPI for RI/pressure) ensures relevance across multiple battery technologies. This adaptability positions the EC-PCF sensor as a versatile platform for next-generation energy storage systems beyond conventional LIBs.

### 4.18. Implementation Challenges and Fabrication Considerations

The transition from simulation to practical implementation presents several challenges that must be addressed for successful sensor deployment. Primary among these is the **liquid-core fabrication process**, which requires specialized fusion splicing techniques to integrate the chirped FBG and EFPI cavity within the EC-PCF while maintaining hermetic seals to prevent electrolyte leakage. The infiltration of high-purity EC into the PCF’s microstructured air holes demands precise pressure and vacuum control to achieve complete, bubble-free filling [27].

- **Thermal expansion mismatch** between the silica fiber, EC filler, and battery components must be carefully managed to avoid mechanical failure during temperature cycling. The coefficients of thermal expansion (CTE) for silica (0.55 ppm/°C), EC (∼20 ppm/°C), and typical battery materials (∼24 ppm/°C) create complex stress states that could affect long-term reliability [22].
- **Electrochemical compatibility** represents another critical challenge. While ethylene carbonate provides inherent compatibility with LIB electrolytes, prolonged exposure to electrochemical potentials and reactive species could potentially degrade the fiber coatings or interface materials. Accelerated aging tests under realistic battery operating conditions are necessary to validate long-term stability.
- **Integration into commercial battery designs** requires minimal intrusion and robust electrical isolation. The 125 μm diameter fiber must be positioned to maximize sensing capability while minimizing impact on battery performance. Embedding strategies must consider electrode stacking processes, winding tension, and potential short-circuit risks.
- **Multiplexing capability** for battery pack monitoring necessitates developing array architectures with multiple sensing points along a single fiber. This requires advanced grating writing techniques and signal processing algorithms to distinguish between closely spaced sensors.

Despite these challenges, the simulation results provide strong justification for continued development. The predicted performance metrics, validated against both numerical models and experimental data, demonstrate sufficient potential to warrant addressing these implementation hurdles. The following development phase should focus on prototype fabrication, accelerated aging tests, and integration studies to transition this promising technology from simulation to practical application.

## 5. Conclusions

This research presented a comprehensive simulation framework for a novel multifunctional optical fiber sensor, establishing a foundational model for advanced battery health monitoring. The key contributions of this computational study include the following:

- **A novel simulated sensor design**: A meticulously modeled Ethylene Carbonate-filled Photonic Crystal Fiber (EC-PCF) design that optically matches the battery electrolyte, maximizing light–matter interaction for enhanced refractive index sensitivity.
- An advanced multiplexed simulation architecture: A robust computational framework integrating a chirped fiber Bragg grating (FBG) and an extrinsic Fabry–Pérot interferometer (EFPI) to enable the simultaneous, decoupled measurement of temperature, strain, and refractive index.
- **A fabrication-resilient modeling approach**: A high-fidelity simulation incorporating Monte Carlo analysis with Sobol sequences to quantify performance robustness against real-world manufacturing tolerances and environmental noise.

The simulation results showed that the proposed sensor achieves high sensitivities—approximately 12.00 pm/°C (temperature), 1.10 pm/με (strain), and 1200 nm/RIU (refractive index)—with exceptionally low cross-talk, demonstrating its potential for accurately reconstructing coupled parameters under dynamic battery operating conditions. The single-fiber design offers significant advantages for future embedded deployment, including minimal intrusion, electromagnetic immunity, and inherent electrolyte compatibility.

**Primary Challenges and Transition to Realization**: While this model provides a high-confidence digital twin of the sensor, its physical realization presents distinct engineering hurdles. The primary challenge involves developing specialized fabrication techniques, including the hermetic sealing of the ethylene carbonate-filled fiber to prevent leakage and the precise integration of the FBG and EFPI components. These manufacturing complexities are the critical next step toward commercial viability.

**Future Directions**:

- **Experimental validation and prototyping**: Physical fabrication of the sensor and in operando validation through integration into 18,650 and pouch cell prototypes under realistic charge–discharge cycling conditions.
- **Multi-sensor network simulation**: Expanding the model to simulate distributed sensor arrays for monitoring cell-to-cell variations within full battery packs.
- **AI-enhanced prognostics**: Integrating machine learning with the multi-parameter output for predictive failure analysis and early detection of degradation signatures.
- **Miniaturization for next-generation batteries**: Development of micro-structured variants compatible with solid-state battery architectures.

In summary, this work establishes a rigorous computational paradigm for optical battery sensing, transitioning the field from external observation to modeled embedded electrochemical interrogation. By providing a tool to simulate correlated insights into coupled failure mechanisms, it opens pathways for the future development of fail-safe battery management systems capable of predicting critical events like thermal runaway. The promising simulation results presented herein form a solid foundation for subsequent experimental development and validation.

## Figures and Tables

**Table 1:** PCF geometry and material parameters.

Parameter	Nominal Value	Tolerance	Rationale
Core diameter	6 μm	±0.1%	Ensures single-mode operation [21]
Pitch (Λ)	4 μm	±0.1%	Balances confinement and fabrication [3]
Air-hole diameter (*d*)	1.5 μm	±0.1%	Achieves fill factor d/Λ=0.375 [3]
Fill factor (d/Λ)	0.375	±1%	Optimizes RI sensitivity; tighter due to EC infiltration [3,15]
Eccentricity	0–0.05	N/A	Models fabrication offset [15]
Cladding diameter	14 μm	N/A	Standard for PCF [21]
Fiber length	140 mm	N/A	Suitable for LIB integration [3]
Silica RI	1.444 (1550 nm, 25 °C)	N/A	Sellmeier equation [18]
EC RI	1.43 (1550 nm, 25 °C, >99% purity)	N/A	LIB electrolyte [3,20]
Silica thermo-optic coefficient	$1.1\times 10^{-5}$/°C	N/A	Yields ∼12 pm/°C sensitivity [17]
EC thermo-optic coefficient	$-4.32\times 10^{-4}$/°C	N/A	Enhances RI sensitivity [3,20]
EFPI thermo-optic coefficient	$-4.0\times 10^{-4}$/°C	N/A	Models cavity RI changes [2]
Silica thermal expansion	$5.5\times 10^{-7}+10^{-10}{\left(T-25\right)}^{2}$/°C	N/A	Minimizes errors [17]
EC thermal expansion	$2\times 10^{-6}+10^{-8}{\left(T-25\right)}^{2}$/°C	N/A	Models EC expansion [3]
Photoelastic coefficients	$p_{11}=0.121,p_{12}=0.270\pm 0.01\times \frac{\lambda -1550\times 10^{-9}}{10^{-9}+10^{-5}\left(T-25\right)}$	N/A	Strain-induced RI for TE/TM modes [23]
Poisson’s ratio	0.17	N/A	Silica property [23]
Young’s modulus	73 GPa	N/A	Silica mechanical property [23]
Bond stiffness	$10^{7}$ N/m	N/A	Strain transfer [2]
Density	2203 kg/m^3^	N/A	Silica [17]
Specific heat	740 J/(kg·K)	N/A	Silica [17]
Heat transfer coefficient	10 W/(m^2^·K)	N/A	Lumped-capacitance model [3]

**Table 2:** FBG parameters.

Parameter	Nominal Value	Tolerance	Rationale
Grating length	10 mm	N/A	Balances sensitivity and compactness [14]
Nominal period ($\Lambda_{0}$)	535 nm (25 °C)	N/A	Centers reflection at ∼1550 nm [11]
Chirp rate	$1.3\times 10^{-4}$ m/m	N/A	Yields ∼2 nm bandwidth [24]
Index modulation (δn)	$3\times 10^{-4}$	N/A	Enhances peak strength [11]
Apodization	Gaussian, $\sigma =L_{\mathrm{grating}}/2$	N/A	Suppresses side-lobes [24]

**Table 3:** EFPI Parameters.

Parameter	Nominal Value	Tolerance	Rationale
Cavity length	20 μm	N/A	High fringe visibility [8]
Reflectivity ($R_{\mathrm{FPI}}$)	0.04	±0.002	Minimizes losses [25]
Phase offset	0–2π (randomized)	N/A	Models interference [11]

**Table 4:** Cross-sensitivity values.

Mode	Cross-Sensitivity (pm/°C·με)	Rationale
TE	0.008	Low interference ensured decoupling [16]
TM	0.007	Minimal birefringence effect [23]
EFPI	0.01	RI response dominated; low thermo-mechanical coupling [8]

**Table 5:** Comparative sensitivity metrics of the proposed sensor versus literature values.

Parameter	This Work	Literature Value	References
Temperature Sensitivity (TE mode)	12.0 pm/°C	10–14 pm/°C	[11,14]
Temperature Sensitivity (TM mode)	11.8 pm/°C	10–14 pm/°C	[11,14]
Strain Sensitivity (TE mode)	1.10 pm/με	1.0–1.3 pm/με	[11,14]
Strain Sensitivity (TM mode)	1.08 pm/με	1.0–1.3 pm/με	[11,14]
RI Sensitivity (EFPI)	1200 nm/RIU	650 nm/RIU	[8,25]

**Table 6:** Comprehensive performance comparison of LIB monitoring sensors.

Sensor Type	Temp. Sens. (pm/°C)	Strain Sens. (pm/με)	RI Sens. (nm/RIU)	Cross- Sensitivity	Multi- Parameter	Ref.
EC-PCF FBG/EFPI (This Work)	12.00 (TE) 11.80 (TM)	1.10 (TE) 1.08 (TM)	1200.00 (EFPI)	0.008 pm/°C·με 0.01 nm/°C·RIU	Yes (T, Strain, RI)	–
Mach-Zehnder (MZI)	70–100	0.5–1.0	50–100	High (T-Strain)	Limited (T, RI)	[21]
Rayleigh Scattering	10–20	0.8–1.2	N/A	Moderate (T-Strain)	No (T, Strain)	[15]
FBG (Standalone)	10–14	1.0–1.3	10–20	Moderate (T-Strain)	Limited (T, Strain)	[14]
EFPI (Standalone)	∼0	∼0	500–800	Low (T-RI)	No (RI)	[8]
Hybrid FBG-EFPI	10–12	1.0–1.2	600–700	Moderate (T-RI)	Yes (T, Strain, RI)	[9]
Plasmonic Fiber-Optic	50–80	N/A	2000–3000	High (T-RI)	Limited (T, RI)	[6]
Brillouin (BOTDA)	1–3 ^a^ (MHz/°C)	0.05–0.1 ^a^ (MHz/με)	N/A	Low (T-Strain)	No (T, Strain)	[19]
Graphene-Coated FBG	15–20	1.2–1.5	100–200	Moderate (T-RI)	Limited (T, Strain, RI)	[18]
Photonic Crystal Waveguide	80–120	N/A	400–600	High (T-RI)	No (T, RI)	[7]

^a^ Brillouin frequency shift units differ from wavelength-based sensors.

## Data Availability

The MATLAB codes used for the simulations, along with the generated .mat and .csv result files, are available and will be uploaded to a public GitHub repository upon publication of this article or upon request. For access to the repository or further inquiries, please contact the corresponding author.

## Abbreviations

LIB	Lithium-Ion Battery
EC-PCF	Ethylene Carbonate-filled Photonic Crystal Fiber
FBG	Fiber Bragg Grating
EFPI	Extrinsic Fabry–Pérot Interferometer
RI	Refractive Index
TE	Transverse Electric (polarization mode)
TM	Transverse Magnetic (polarization mode)
TMM	Transfer Matrix Method
PCF	Photonic Crystal Fiber
BMS	Battery Management System
PDL	Polarization Dependent Loss
ODE	Ordinary Differential Equation
SNR	Signal-to-Noise Ratio
RMSE	Root Mean Square Error
EV	Electric Vehicle
T	Temperature
ϵ	Strain
Ø	Diameter
